# Maturation of the preterm gastrointestinal tract can be defined by host and microbial markers for digestion and barrier defense

**DOI:** 10.1038/s41598-021-92222-y

**Published:** 2021-06-17

**Authors:** Jannie G. E. Henderickx, Romy D. Zwittink, Ingrid B. Renes, Richard A. van Lingen, Diny van Zoeren-Grobben, Liesbeth J. Groot Jebbink, Sjef Boeren, Ruurd M. van Elburg, Jan Knol, Clara Belzer

**Affiliations:** 1 Laboratory of Microbiology, Wageningen University and Research, Stippeneng 4, 6708 WE Wageningen, The Netherlands; 2 Center for Microbiome Analyses and Therapeutics, Leiden University Medical Center, Leiden, The Netherlands; 3 Danone Nutricia Research, Utrecht, the Netherlands; 4 Emma Children’s Hospital, Amsterdam UMC, Location AMC Amsterdam, Amsterdam, The Netherlands; 5 Department of Neonatology, Isala Women and Children’s Hospital, Zwolle, The Netherlands; 6 Laboratory of Biochemistry, Wageningen University and Research, Wageningen, The Netherlands; 7 Emma Children’s Hospital, Amsterdam UMC, University of Amsterdam, Amsterdam, The Netherlands

**Keywords:** Neonatology, Preterm birth, Microbiome, Large intestine, Microbiota, Stomach, Paediatric research, Nutrition

## Abstract

Functionality of the gastrointestinal tract is essential for growth and development of newborns. Preterm infants have an immature gastrointestinal tract, which is a major challenge in neonatal care. This study aims to improve the understanding of gastrointestinal functionality and maturation during the early life of preterm infants by means of gastrointestinal enzyme activity assays and metaproteomics. In this single-center, observational study, preterm infants born between 24 and 33 weeks (n = 40) and term infants born between 37 and 42 weeks (n = 3), who were admitted to Isala (Zwolle, the Netherlands), were studied. Enzyme activity analyses identified active proteases in gastric aspirates of preterm infants. Metaproteomics revealed human milk, digestive and immunological proteins in gastric aspirates of preterm infants and feces of preterm and term infants. The fecal proteome of preterm infants was deprived of gastrointestinal barrier-related proteins during the first six postnatal weeks compared to term infants. In preterm infants, bacterial oxidative stress proteins were increased compared to term infants and higher birth weight correlated to higher relative abundance of bifidobacterial proteins in postnatal week 3 to 6. Our findings indicate that gastrointestinal and beneficial microbial proteins involved in gastrointestinal maturity are associated with gestational and postnatal age.

## Introduction

Preterm birth interrupts the natural, intrauterine development of the gastrointestinal tract (GIT), immune system and microbiota that occurs during the third trimester^1,2^. The GIT continues to develop after birth with the environment deviating from mother’s womb. Early exposure to a deviating environment affects the infant and maturation processes on a systemic level.

Strict feeding regimens are implemented to orchestrate optimal maturation of the GIT, which is crucial for infant growth, development and health. Human milk is the first choice for both term and preterm infants as it has nutritious, immunomodulatory and microbial benefits^3^. Whether nutritional components of human milk can be absorbed and digested largely depends on gastrointestinal maturity of the infant^4^. Some digestive enzymes and gastrointestinal motility functions develop during later stages of gestation, leading to suboptimal functioning upon preterm birth^4,5^. This affects proteolysis of major milk proteins in preterm infants for example^6^, even though it still occurs^7^. Incomplete breakdown of proteins can be beneficial or harmful, depending on which proteins remain intact^8^. Additionally, underdeveloped gastrointestinal motility may lead to nutrient retention that could initiate a sequence of events with translocation of microbes or their toxic products as a consequence^9^.

Gastrointestinal maturity additionally plays an important role in gastrointestinal barrier functioning, which is crucial for maintaining gastrointestinal homeostasis and infant health^10^. Preterm infants have a leaky gut in the first weeks of life as the intestinal barrier develops^11–13^. Along with gestational age, multiple factors affect intestinal permeability in preterm infants, including infection, inflammation, feeding type and antibiotic exposure^14–16^.

Host-microbe interactions occur at the gut barrier and impact physiological development of the GIT, the immune system and human milk digestion^2,17^. Preterm infants are particularly susceptible to sepsis and necrotizing enterocolitis (NEC) due to immaturity of the intestinal epithelial barrier, the immune system as well as their microbiota development^9,12,18^.

Metaproteomics offers great potential to functionally characterize organisms and is increasingly used to supplement compositional profiling of the human microbiome^19–23^. This study aims to improve the understanding of gastrointestinal functionality and maturation during the early life of preterm infants by means of gastrointestinal enzyme activity assays and metaproteomics. Here, we add new enzyme activity analyses to previously acquired metaproteomics data^24^. Moreover, gastric aspirates and term infants were newly added to the metaproteomics analyses, which focus on human proteins and their implications for interactions with previous findings on the microbiota^24^.

## Results

### Metaproteomic characterization

**Figure 1:**
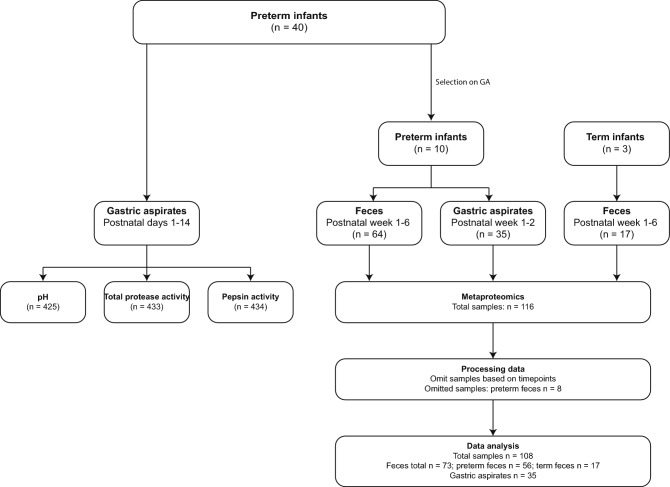
Overview and workflow of this study. Preterm and term infants were part the EIBER study. Of forty preterm infants, gastric aspirates were collected during postnatal days 1–14 (left). From ten out of forty preterm infants, feces were additionally collected in postnatal week 1-6 with the exception of week 5; gastric aspirates of the first two postnatal weeks were included for metaproteomics if they were collected on similar timepoints as the fecal samples. Three term infants were included as healthy reference. Feces of those infants were collected in postnatal week 1–6 with the exception of week 5. GA: gestational age.

740,343 MS/MS spectra were recorded of which 89,294 were identified. After omitting samples (Fig. 1), metaproteomics generated 11,885 unique peptides and 3181 protein groups in 108 samples. 2317 protein groups remained after protein filtering. Of the identified protein groups, 886 proteins were human- and/or bovine-derived and 1431 proteins were bacterial-derived. On average, 1409 ± 456.6 unique peptides and 206.3 ± 66.5 protein groups were detected in a sample. 199.3 ± 62.8 and 243.8 ± 74.6 protein groups were detected on average in preterm and term infants, respectively.

### Gastric proteases and peptidases are present and active in the preterm gastric proteome

Forty preterm infants were part of this study. Of all these infants, gastric aspirates were collected during postnatal days 1–14 (Fig. 1). These samples were used for pH measurements, total protease activity and pepsin activity. At some timepoints, samples were unavailable or insufficient in volume to conduct all measurements, resulting in 425 samples for pH measurement, 433 for total protease activity assays and 434 for pepsin activity assays (Fig. 1).

**Figure 2:**
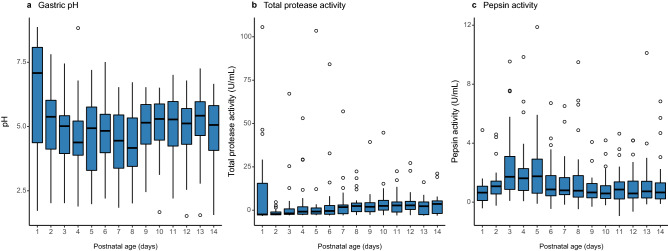
Gastric pH and enzyme activity during the first two postnatal weeks of preterm infants. Dynamics of (**a**) gastric pH, (**b**) total protease activity and (**c**) pepsin activity. Boxplots show the median, 25th and 75th percentiles, and minimal and maximal values with the exception of outliers (circles, lower or higher than 1.5 * inter-quartile range).

Median gastric pH of preterm infants fluctuated between 4.5 and 5.5 over time (Fig. 2A). In ten out of forty infants, the gastric pH was exceptionally high (> 8.0 pH) at day of birth but mean gastric pH did not differ significantly from the other infants in the days thereafter (*p* > 0.99). Intra-individual differences were high, with a mean difference of 4.2 ± 1.3 (s.d.) between the lowest and highest pH measured during the first six weeks of life. Total protease and pepsin activity showed high variation between and within infants. While median total protease activity was higher in the second than the first postnatal week, pepsin activity remained relatively stable (Fig. 2B,C). Being a pH-dependent enzyme, pepsin activity decreased with higher gastric pH and was not affected by postnatal age (ρ = −0.32, *p* = 1.3 × 10^−11^). Interestingly, pepsin was not detected in the gastric proteome by means of LC-MS/MS. However, other proteases, like trypsin and chymotrypsin-like elastase family members 2A, 3A and 3B could be identified.

### Human and microbial proteins across the gastrointestinal tract

Ten out of the forty preterm infants were selected for metaproteomics based on gestational age (Fig. 1, Supplementary Table S1)^24^. Additionally, three term infants from the EIBER study were included as reference. From all these infants, fecal samples (n = 81) were collected right after birth and at postnatal weeks one, two, three, four and six. Sixty-four fecal samples derived from the ten preterm infants and 17 from the three term infants. Gastric aspirates collected from the ten preterm infants during the first two postnatal weeks were included for metaproteomics if they were collected on similar timepoints as the fecal samples (n = 35). Eight fecal samples from preterm infants were collected in between the intended timepoints and were therefore omitted before data analysis, leaving a total of 56 fecal samples of preterm infants (Fig. 1).

#### Specific human milk proteins resist degradation in the preterm gastrointestinal tract

**Figure 3:**
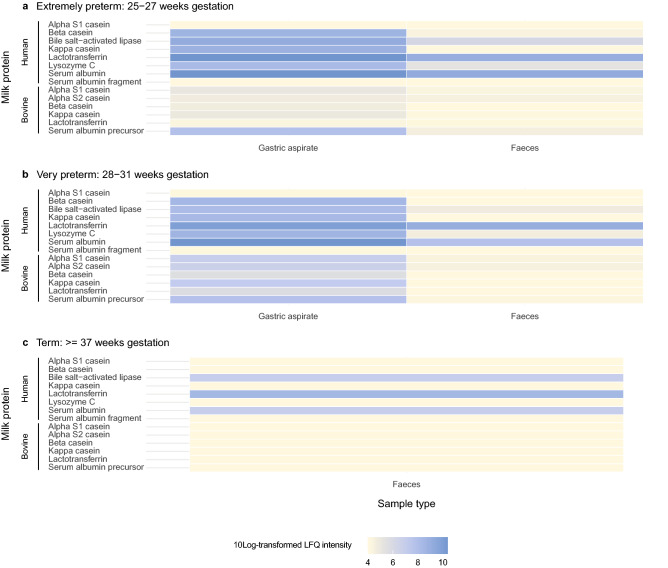
Normalized abundance of milk-derived proteins of human and bovine source in gastric aspirates and feces during the first two postnatal weeks. Milk-derived proteins in gastric aspirates and feces of (**a**) extremely preterm, (**b**) very preterm and (**c**) term infants. Log10 transformed LFQ values are shown. Coloring is based on abundance from least abundant (yellow) to most abundant (blue).

In addition to the presence of proteases, milk-derived proteins were present in gastric aspirates and feces of preterm infants throughout the first two postnatal weeks. These included bile-salt activated lipase, lactotransferrin, caseins, alpha-lactalbumin and serum albumin (Supplementary Table S2). In the gastric aspirates of extremely preterm infants in the first two postnatal weeks, more than 30.0% of identified human milk proteins consisted of casein fragments. In feces, only 0.07% and 0.2% of identified human milk proteins were casein fragments in week one and two respectively. In contrast to extremely preterm infants, the relative abundance of casein fragments in gastric aspirates of very preterm infants was higher with 48.1% and 47.5% in week one and two respectively. No casein fragments were detected in feces. Human milk-derived lactotransferrin and serum albumin were also detected in fecal samples of all preterm and term infants, while no bovine-derived proteins were observed (Fig. 3 and Supplementary Fig. S1).

#### Birth weight positively correlates to bifidobacterial protein abundance in preterm infants from the third postnatal week onwards

In term infant’s feces, relative abundance of bacterial proteins gradually increased from 18% to 34% over the first six weeks, while the abundance of host- and dietary-derived proteins decreased. The ratio bacterial to eukaryote proteins developed more stochastically in preterm infants (Supplementary Fig. S2). The bacterial proteins abundance in extremely preterm infants was 6% and remained significantly lower than that of term infants up till the end of the six weeks (*p* = 0.04). For very preterm infants, bacterial protein abundance increased to 31%, reaching similar levels as that of term infants in week 6. Moreover, extremely preterm infants were characterized by a low mean relative abundance of bifidobacterial-derived proteins fluctuating between 6% and 10% throughout the six-week period (Supplementary Fig. S3). In contrast, bifidobacterial-derived proteins reached proportions as high as 41% and 52% in the sixth week of very preterm and term infants, respectively.

Weight parameters were correlated to relative abundance of bifidobacterial-derived proteins in preterm infant’s feces. For term infants, no data on weight gain was available. For preterm infants, higher birth weight was significantly correlated to higher proportions of bifidobacterial proteins from week 3 onwards (ρ > 0.75 and *p* < 0.05) (Supplementary Fig. S4). In contrast, higher *Bifidobacterium* relative abundance did not significantly correlate to growth velocity during the first six postnatal weeks (*p* ≥ 0.23) (Supplementary Fig. S5).

#### Gastrointestinal barrier-related proteins are less abundant in preterm infant’s feces

**Figure 4:**
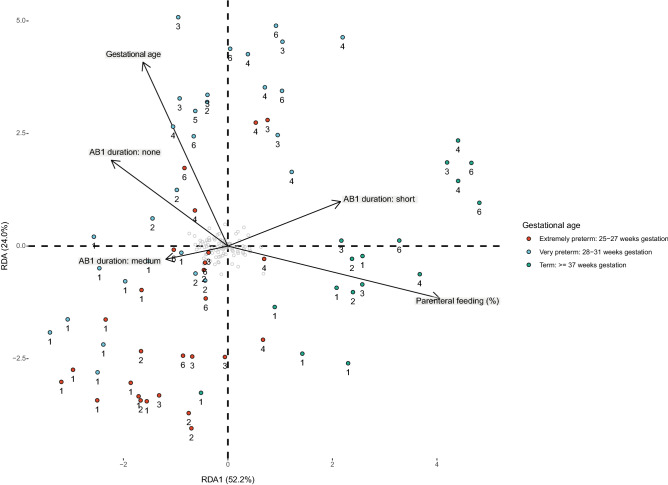
Redundancy analysis on the fecal proteome of preterm and term infants during the first six postnatal weeks. Clinical variables shown by arrows significantly explain variation in the proteome as selected by forward and reverse automatic stepwise model selection. Colored points indicate infant samples of one timepoint, numbers indicate the corresponding postnatal week and grey points indicate centroids of identified proteins. AB1: first antibiotic treatment including no treatment and treatment of short (< 3 days) or medium (3–5 days) duration.

**Figure 5:**
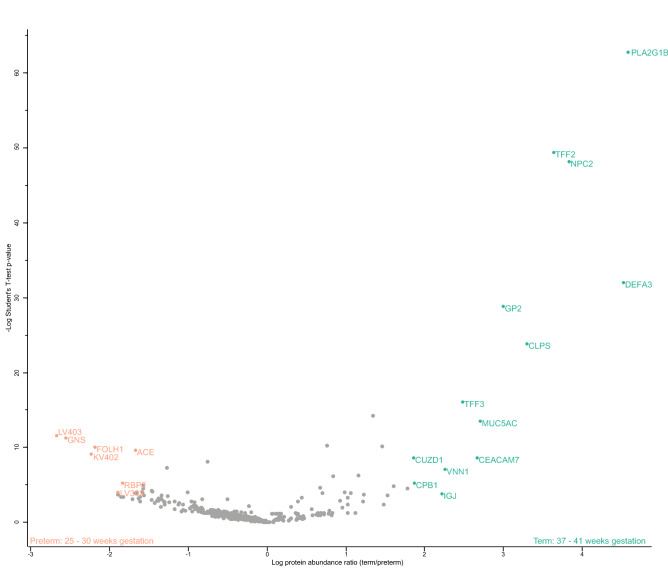
Volcano plot showing differences within the fecal proteome of preterm and term infants. LFQ intensities of fecal samples from preterm (n = 64) and term (n = 17) infants were used to generate the volcano plot in Perseus version 1.6.2.1^56^. LFQ intensities were 10log transformed, samples were assigned to its designated study group and compared with a Student’s two-sample t-test with permutation-based FDR correction. P-values as indicated on the y-axis are -10log transformed. The differentially expressed proteins are marked by gene names. Upregulated proteins in preterm infants include: LV403: Ig lambda chain V-IV region Hil; GNS: Glucosamine (N-acetyl)-6-sulfatase; FOLH1: Glutamate carboxypeptidase 2; ACE: Angiotensin-converting enzyme; KV402: Ig kappa chain V-IV region Len; RBP2: Retinol-binding protein 2 (fragment); LV302: Ig lambda chain V-II region LOI. Upregulated proteins in term infants include PLA2G1B: Phospholipase A2; TFF2: Trefoil factor 2; NPC2: Epididymal secretory protein E1 (fragment); DEFA3: Neutrophil defensin 3; GP2: Pancreatic secretory granule membrane major glycoprotein GP2 (fragment); CLPS: Colipase; TFF3: Trefoil factor 3; MUC5AC: Mucin-5AC; CUZD1: CUB and zona pellucida-like domain-containing protein 1; CEACAM7: Carcinoembryonic antigen-related cell adhesion molecule 7; VNN1: Pantetheinase; CPB1: Carboxypeptidase B; IGJ: Immunoglobulin J chain (fragment).

**Figure 6:**
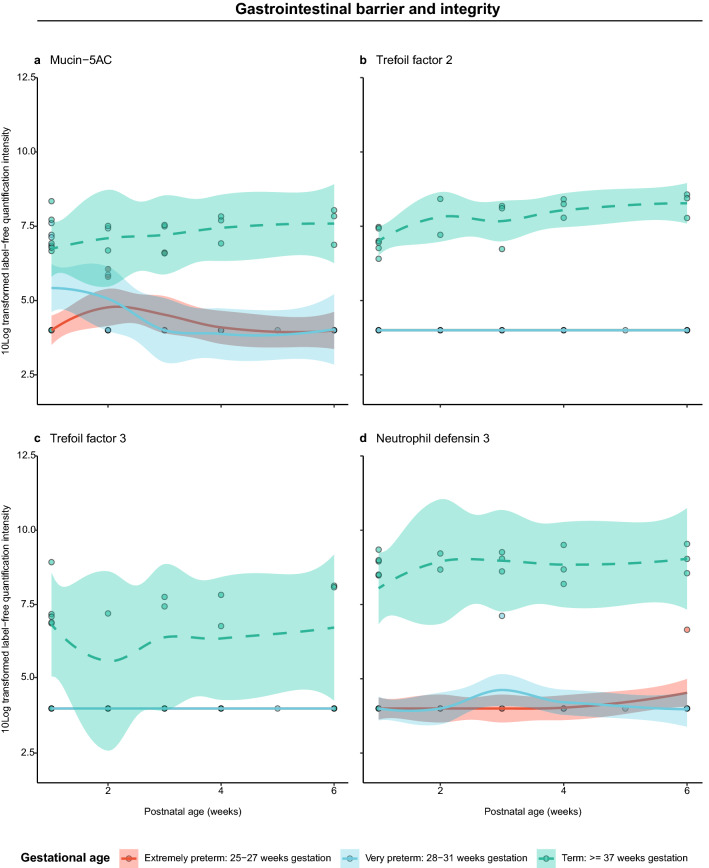
Temporal dynamics of gastrointestinal integrity and maturation markers in the fecal proteome of preterm and term infants. (**a**) Mucin-5AC, (**b**) trefoil factor 2, (**c**) trefoil factor 3 and (**d**) neutrophil defensin 3. The y-axis shows 10Log transformed label free quantification (LFQ) intensity. The x-axis shows the postnatal age in weeks for each gestational age group.

Based on redundancy analysis, gestational age was identified as significant driver for differences in the fecal proteome of preterm and term infants during the first six postnatal weeks (Fig. 4, Supplementary Table S3). Twelve (out of 804) human-derived proteins’ abundances were significantly lower in preterm infants’ feces compared to term infants’ feces during the first six postnatal weeks (Fig. 5, Supplementary Table S4). These proteins included barrier and innate mucosal immune proteins mucin-5AC (MUC5AC), trefoil factor 2 (TFF2), trefoil factor 3 (TFF3) and neutrophil defensin 3 (DEFA3) and proteins involved in lipid metabolism. As such, these proteins were further analyzed longitudinally by temporal dynamic patterns. MUC5AC showed a 2.7-fold change in term infants compared to preterm infants (Fig. 5) and was detected in low levels of preterm infants during the first two postnatal weeks (Fig. 6A). From the third week onwards, MUC5AC intensity decreased further in preterm infants while it increased in term infants. From the third postnatal week onwards, MUC5AC levels were significantly higher in term infants compared to that of preterm infants (*p* < 0.05). A similar pattern was observed for TFF2 (Fig. 6B) with a 3.6-fold change difference between term infants and preterm infants (Fig. 5). In contrast to term infants, TFF3 was not detected in preterm infants during the first six weeks of life (Fig. 6C). TFF3 had a 2.5-fold change in term infants compared to preterm infants (Fig. 5). DEFA3 was significantly higher in term infants compared to preterm infants during the whole period six-week period (*p* < 0.05) (Fig. 6D) and reached 4.5-fold change difference in term infants compared to preterm infants (Fig. 5).

#### Preterm infants express higher levels of microbial oxidative stress proteins compared to term infants

Our previous work indicated that delayed colonisation by beneficial, strict anaerobes was associated with respiratory support strategies^24^. Samples with low levels of bifidobacterial proteins showed relatively high levels of proteins derived from opportunistic pathogens including *Enterococcus* spp., *Escherichia* spp. and *Klebsiella* spp. (Supplementary Fig. S3). Being facultative anaerobic bacteria, expression of oxidative stress proteins may provide competitive advantage. These genera expressed oxidative stress proteins at different levels in the gestational age groups. Extremely preterm infants, characterized by a low mean relative abundance of bifidobacterial-derived proteins, had significantly higher levels of oxidative stress proteins compared to term infants in the second till fourth postnatal week (*p* ≤ 0.01) (Supplementary Fig. S6).

#### Human digestive and immunological proteins are consistently present in the preterm infant’s gastrointestinal tract

Many bioactive proteins were consistently identified in preterm infants. For example, immunoglobulin structures and other innate immune proteins were attributes with highest contribution to variation in the gastric proteome by principal component analysis (Supplementary Fig. S7) as well as by comparison of fecal protein abundances in preterm and term infants (Fig. 5, Supplementary Table S4). Immunoglobulin structures that were significantly more abundant in preterm infants included Ig lambda chain V-III region LOI, Ig kappa chain V–IV region Len and Ig lambda chain V–IV region Hil (−1.9, −2.2- and −2.7-fold change respectively between preterm and term infants). Additionally, human catabolic enzymes were more abundant in preterm infants (Fig. 5, Supplementary Table S4) including angiotensin-converting enzyme, glutamate carboxypeptidase 2 and N-acetylglucosamine-6-sulfatase (−1.7-, −2.2- and −2.6-fold change respectively between preterm and term infants). Apart from significant differences, a big variety of human-derived proteins involved in digestion and immune responses were consistently identified in both gastric and fecal proteomes of preterm infants (Supplementary Table S5).

## Discussion

Our findings show that preterm infants express enzymes for human milk protein degradation, albeit to a lesser extent than term infants. Digestion likely starts in the gastric environment with proteases derived from mother and/or infant. Moreover, the gastrointestinal barrier of preterm infants is impaired during the first six postnatal week together with less milk-degrading microbes and more bacterial oxidative stress proteins. Although digestive enzymes and gastrointestinal permeability are known to be suboptimal in preterm infants, our findings address these issues for the first time by combining the proteomic profiles of infant gastric aspirates and feces. Other metaproteomic studies in preterm infants have addressed functionality of the microbiome^21,22^. Additionally, we have added a host and developmental perspective to this by monitoring in the first six postnatal weeks.

Similar to findings by Omari *et al.*^25^, we found average gastric pH fluctuated during the first two postnatal weeks. Extremely high gastric pH at day of birth, as observed in some infants in our study, might be due to swallowing of alkaline amniotic fluid^26^. Median pepsin activity was relatively stable, yet very low. High pH combined with low pepsin activity could affect gastric digestive capacity of preterm infants and thereby decrease their nutrient utilization potential^6,8^. Although enzyme activity analyses showed pepsin was active in the stomach, we could not identify pepsin in the gastric proteome by means of LC-MS/MS. Yet, our results suggest preterm infants are equipped to degrade human milk proteins, as we identified proteases, peptidases and various other digestive enzymes in the gastric and fecal proteome. The activity of these enzymes depends on the maturation status of the infant and may thereby introduce variation in the protein groups identified in each infant, as was shown by our high protein identity variety across samples. The identified enzymes could also be derived from human milk^27,28^. A previous study, however, showed that human milk-derived proteases cannot compensate for the low gastric protein digestion capacity observed in preterm infants^6^. In agreement with our findings, other studies have shown lower proteolytic capacity of gastric enzymes for human milk proteins in preterm infants compared to term infants^6^. Milk peptides, including caseins, can survive gastrointestinal digestion^29^, which we could confirm in our study in a quantitative and longitudinal manner. As milk peptides are an important source of peptides and amino acids for rapidly growing infants^30^, the impaired degradation and/or absorption of human milk proteins in preterm infants could have serious consequences on energy acquisition and subsequent growth in early life. However, providing infants with protein hydrolysates did not improve growth and weight gain^31^. The microbiota composition in preterm infants may further influence metabolic activities as shown by other metaproteomic studies in preterm infants that identified microbiota-associated metabolic shifts^21,22^. In our previous work, we similarly have shown that a *Bifidobacterium*-dominated community, as observed in very preterm but not in extremely preterm infants, is associated with increased bacterial proteins involved in carbohydrate and energy metabolism^24^. Here, we have described a correlation between bifidobacterial proteins and birth weight in preterm infants. Infants with higher birth weight are less likely to encounter complications and are more likely to have better early neonatal circumstances compared to low-birth-weight infants. Less and shorter antibiotic treatments as well as less respiratory support might allow beneficial obligate anaerobes, such as bifidobacteria, to thrive. Delayed colonization of such bacteria was observed with more and longer antibiotic treatments as well as with increased duration of respiratory support^24,32,33^. As a consequence of a higher birth weight, maturation of the GIT as well as bifidobacterial abundance would be promoted, further supporting human milk utilization capacity of the microbiota and weight gain. Potential associations between microbiota and weight gain should be further investigated in a larger cohort of preterm infants.

In addition to digestion, we have shown impaired levels of gastrointestinal barrier-related proteins in preterm infants over the first six postnatal weeks. While MUC5AC is a gel-forming glycoprotein lining gastric and respiratory tract epithelia, trefoil factors are mucin-associated peptides involved in protection and repair of the gastrointestinal mucosa by being involved in restitution and stimulation of immunocyte migration^34–36^. Interaction of TFF2 or TFF3 with MUC5AC has not been reported so far but is likely due to proven interactions between trefoil factor 1 and MUC5AC as well as homology within a conserved trefoil domain^37^. Therefore, our findings could indicate a less thick and stable mucus layer in the GIT of preterm infants that could subsequently impair the intestinal barrier as described previously^11^. Other metaproteomic studies in preterm infants identified proteins related to gut mucosal barrier development and protection, including MUC5AC and trefoil factors^21,22^. Our results showed an inverse association of gastrointestinal barrier-related proteins with bacterial oxidative stress proteins of facultative anaerobes. In our previous work, delayed colonisation by beneficial, strict anaerobes was associated with respiratory support strategies including ventilation and/or continuous positive airway pressure (CPAP)^24^. Hence, we hypothesized that respiratory support might introduce oxygen into the lumen. Subsequently, the aerobic environment could decrease abundance of beneficial, strict anaerobic microbes such as *Bifidobacterium* spp., that produce short-chain fatty acids involved in the production of anti-inflammatory cytokines and stimulation of the intestinal barrier function^16,38–40^. This in turn might sustain an aerobic environment in which facultative anaerobes such as *Enterococcus* spp. and *Klebsiella* spp. benefit from oxygen while restraining oxidative stress^41^. While *Bifidobacterium* may protect against intestinal barrier dysfunction^42^, products of *Enterococcus* could compromise the intestinal epithelial barrier^43^. Our previous work on the same cohort indicated a delay in bacterial colonization in extremely preterm infants compared to infants born at later gestational ages, as well as decreased abundance of *Bifidobacterium*-derived proteins^24^, suggesting that this gestational age category is particularly prone to an impaired intestinal barrier and a leaky gut.

Digestive and immune proteins were consistently identified in the gastric and fecal proteome. Proteins related to innate immune responses, including immunoglobulins and antibacterial proteins, have previously been identified in preterm infants^21,22^. It remains unknown whether these proteins are active and whether they are produced by the preterm infant itself or derived from human milk, even though many proteins have been detected in human milk^27,28,44,45^ and preterm infants are immunocompromised^46^. Some bioactive proteins are more evident to derive from human milk, such as secretory immunoglobulin A^47^. Other bioactive proteins that we identified in feces include casein fragments, lactotransferrin and serum albumin, as described previously^48^. By surviving gastrointestinal digestion, these components could confer functional properties that could protect against neonatal sepsis and NEC^49^ although certainty of evidence in these cases is low^50^. Human milk, thus, acts at the gut-barrier interface where it supports functional development of the GIT, shapes the microbiome and positively influences health outcomes^51,52^.

While our findings indicate impaired digestion and gastrointestinal barrier defense in preterm infants, we acknowledge the relatively small number of particularly term infants described in this study. As the main objective of this pilot study was to elucidate metaproteomes of preterm infants, few term infants were recruited. This should be taken into account when interpreting the data. Moreover, metaproteomics of the human gut has its challenges^20^. Future studies should focus on increasing depth and coverage of the microbiome, sample preparation throughput and multiplexity of MS measurements. Moreover, technical barriers for bioinformatic data processing require additional efforts^19,20^.

## Conclusion

Our findings indicate that gastrointestinal and beneficial microbial proteins involved in gastrointestinal maturity are associated with gestational and postnatal age. While digestive enzymes and gastrointestinal permeability are known to be suboptimal in preterm infants, this is the first study measuring both human and microbial proteins in stomach and feces during the first six postnatal weeks. The gut barrier proves to be an important environment where gastrointestinal epithelium, immune system and microbiota interact to drive growth, development and health of the preterm infant. More insights might lead to the design of optimized nutrition support strategies based on the characteristics of the preterm infant and its gut maturation status.

## Materials and methods

### Ethics declaration

The board from the Medical Ethical Committee of Isala Zwolle concluded that this study does not fall under the scope of the Medical Research Involving Human Subjects Act (WMO). Informed consent was obtained from both parents of all individual participants included in the study.

### Study description

The study was part of the EIBER study; a single-center, observational study involving term and preterm infants admitted to the neonatal intensive care unit or the pediatric ward of Isala in Zwolle, The Netherlands. Mothers were encouraged to breastfeed at all times. If needed, infants were supplemented or fed with preterm formula (Nutrilon Nenatal Start, Danone Nutricia, The Netherlands).

As part of the EIBER study, gastric aspirates were obtained on a daily basis during the first 14 days of life in all preterm infants having a nasogastric tube on clinical grounds (Fig. 1, gestational age of 24-33 weeks, n = 40 infants). Enteral feeding was started as soon as possible with gradual increments but was supplemented with parenteral nutrition if necessary. Samples of this part were used to perform pH measurements and enzyme activity analyses.

Another part of the EIBER study included gastric aspirate and fecal sample collection immediately after birth and at postnatal weeks one, two, three, four and six. This part included both preterm and term infants that were selected based on gestational age (Fig. 1). Out of the forty preterm infants, ten preterm infants were selected based on a gestational age < 32 weeks, whereas three term infants were selected based on a gestational age between 37 and 41 weeks. The amount of collected gastric aspirate was roughly 1 mL; the minimal amount of collected feces was one scoop. Samples of this part were used to perform metaproteomics and were used previously as well for 16S rRNA gene amplicon sequencing^24^.

### Subjects and sample selection

#### pH and enzyme activity analyses

From the ten preterm infants (100 samples), as well as thirty additional preterm infants (325–334 samples), aspirates of residual gastric content were collected daily during the first two postnatal weeks (Fig. 1). The thirty additional preterm infants were selected if gastric aspirates of minimally eight timepoints were available. At collection, samples were frozen at −20 °C and stored at −80 °C.

#### Metaproteomics

Ten preterm infants and three term infants from the EIBER study were selected based on gestational age (Fig. 1, Supplementary Table S1)^24^. From all these infants, fecal samples were collected right after birth and at postnatal weeks one, two, three, four and six (n = 81). Sixty-four samples derived from preterm infants and 17 from term infants. On the same timepoints, gastric aspirates were collected from preterm infants (n = 35). The metaproteomes of these infants have been generated and described previously, with the main objective to characterize the bacterial part of the preterm infant’s fecal metaproteomes^24^.

### Enzyme activity analysis

Gastric aspirate samples were thawed on ice, centrifuged (3000 rpm; 4 °C) and the cream layer was removed^53^. pH of the supernatant was determined (n = 425 samples) and samples were centrifuged to remove any remaining cream fraction (14,000 rpm; 4°C)^53^. Total protease (n = 433 samples) and pepsin activity (n = 434 samples) were determined using the green fluorescence EnzChek Protease Assay Kit (Molecular Probes, Eugene, OR, USA) in duplicate according to manufacturer’s instructions. For determining total protease activity, 10mM TRIS buffer (pH 7.8) was used and the standard curve was generated using pancreatin from porcine pancreas (Sigma-Aldrich)^53^. For determining pepsin activity, 10mM HCl buffer with pH 2.2 was used and the standard curve was generated using pepsin from porcine gastric mucosa (Sigma-Aldrich)^53^.

### Sample processing for metaproteomic analysis

Extraction of proteins from feces was performed mechanically by repeated bead beating as described previously (n = 81 samples)^24,54^. Gastric aspirates were thawed on ice, centrifuged (3000 rpm; 4 °C) and pellet was removed (n = 35 samples). pH of the supernatant was determined and samples were centrifuged to remove debris (max rpm; 4°C). Fecal and gastric proteins were quantified using Qubit Protein Assay Kit on a Qubit 2.0 fluorometer (Life Technologies, Carlsbad, CA, USA) and diluted in PBS to obtain a 3 μg/μl and 5 μg/μl concentration, respectively. In gel digestion procedures, database construction, analysis of MS/MS spectra and protein grouping with MaxQuant 1.3.0.5^55^ were performed as previously described^24^. In the current study, no additional mapping of initial search results was performed to functionally classify proteins.

### Metaproteomic database construction

The metaproteomic database has been constructed by Zwittink et al.^24^. The in-house database was accommodated to the study group in order to decrease the chance of false identification. Bacterial genera were selected based on their identification in infants by the Human Microbiome Project reference genomes and by 454 pyrosequencing^24^. Selected bacterial genera were retrieved from Uniprot and their proteomes were merged with proteomes of human, cow, *Candida* spp. and common contaminants^24^. In total, 87 bacterial species and 438,537 sequences were captured in the in-house database (contents of the in-house generated protein database are presented in Zwittink et al.^24^). Taxonomic classification of MS/MS spectra was performed until genus level as there was high protein sequence homology among species of the same genus^24^.

### Data transformation

Eight fecal samples of preterm infants were not collected on correct timepoints and were excluded, resulting in 73 fecal samples (n = 56 preterm samples; n = 17 term samples) and 108 samples in total for further analyses. In Perseus version 1.6.2.1^56^, Label-Free Quantification (LFQ) intensities were 10Log transformed. Resulting NaN values were replaced by the value 4.0, which was lower than minimally observed, and further processed as described before^24^. These pre-processed LFQ intensities were used for analyses. Intensity Based Absolute Quantification (iBAQ) intensities were used to determine relative abundances of iBAQ intensities (riBAQ) like described previously^57^.

Growth velocity was calculated based on clinical data according to the exponential model as described by Patel *et al.*^58^:

$$\left[ {{\text{1}}000 \times {\text{ln}}\left( {{\text{W}}_{{\text{n}}} /{\text{W}}_{{\text{1}}} } \right)} \right]/\left( {{\text{D}}_{{\text{n}}} - {\text{D}}_{{\text{1}}} } \right)$$

where W is the weight in grams, D is day; 1 indicates the beginning of the time interval and n is the end of the time interval, in days^58^. In all cases, birth weight was selected as W_1_.

### Statistical analysis

All analyses were performed in R version 3.6.1^59^. In all cases, (adjusted) p-values < 0.05 were considered statistically significant.

Redundancy analysis (RDA) was performed with the *vegan* package version 2.5-6^60^. Missing values of explanatory variables were omitted and LFQ intensities were mean centered. Forward and reverse automatic stepwise model selection for constrained ordination was performed using *ordistep* from the *vegan* package. For robustness, *ordiR2step* was also performed. Both methods corresponded in selection of significant terms. Resulting p-values were adjusted with *p.adjust* from the *Stats* package using FDR correction.

Volcano plots were generated in Perseus version 1.6.2.1^56^. First, distribution of LFQ values was visually inspected per sample for normality. A two-sided Student’s t-test on LFQ intensities of human- and bovine-derived proteins between two groups was performed with an s0 of 1 and a permutation-based FDR with 250 permutations and an FDR of 0.01.

Temporal dynamic plots were generated per protein of interest with the *ggplot2* package version 3.3.0^61^. Default non-parametric LOESS regression was performed with a 95% confidence interval to generate a smooth fitted line. Individual data points were additionally plotted.

Spearman correlations of clinical data to bifidobacterial proteins were performed on riBAQ intensities using the *ggscatter* function of the *ggpubr* package version 0.3.0^62^. Birth weight was not significantly greater in very preterm infants compared to extremely preterm infants based on a one-sided Mann-Whitney U test (*p* > 0.99) and were therefore grouped for correlation analyses. Mean riBAQ intensities were calculated per gestational age category and postnatal week. Individual data points as well as a regression line with a 95% confidence interval were plotted.

For bacterial oxidative stress proteins, mean riBAQ intensities were calculated per gestational age category and postnatal week. Of each sample, the sum iBAQ intensities of listed oxidative stress proteins were divided by the sum iBAQ intensities of all bacterial proteins (Supplementary Table S6). Comparison between gestational age groups in specific postnatal weeks was performed with Dunn’s test.

## Supplementary Information

Supplementary Information.

## Data Availability

The mass spectrometry data have been deposited to the ProteomeXchange Consortium^63^ via the PRIDE partner repository with dataset identifier PXD005574.
