# Supplementary material for: Maturation of the preterm gastrointestinal tract can be defined by host and microbial markers for digestion and barrier defense
Source: Sci Rep. 2021 Jun 17;11:12808. doi: 10.1038/s41598-021-92222-y (PMC8211855; doi:10.1038/s41598-021-92222-y)
Supplement: Supplementary file 1 — Supplementary Information. [file 41598_2021_92222_MOESM1_ESM.pdf]

Supplementary Information

Maturation of the preterm gastrointestinal tract can be defined by host and microbial markers for digestion and barrier defense

Jannie G.E. Henderickx[‡], Romy D. Zwittink[‡], Ingrid B. Renes, Richard A. van Lingen, Diny van Zoeren-Grobben, Liesbeth J. Groot Jebbink, Sjef Boeren, Ruurd M. van Elburg, Jan Knol, Clara Belzer*

Figure S1. Cumulative relative abundance of casein fragments in feces of preterm and term infants during the first six postnatal weeks.

Figure S2. Distribution of proteins derived from human, bovine, human or bovine and bacterial source per postnatal week.

Figure S3. Distribution of proteins derived from bacterial genera *Bifidobacterium*, *Enterococcus*, *Klebsiella* and other genera per postnatal week.

Figure S4. Spearman correlations between preterm infant's relative abundance of *Bifidobacterium*-derived proteins in postnatal weeks 1-6 and birth weight.

Figure S5. Spearman correlations between preterm infant's relative abundance of *Bifidobacterium*-derived proteins in postnatal weeks 1-6 and growth velocity.

Figure S6. Relative abundance of bacterial oxidative stress proteins per postnatal week.

Figure S7. Principal component analysis on the gastric proteome of preterm infants during the first two postnatal weeks.

Table S1. Infant characteristics of two subsets of the EIBER cohort.

Table S2. Differentially abundant human- and bovine-derived proteins between gastric and fecal samples of preterm infants during postnatal weeks one and two.

Table S3. Tables of RDA data.

Table S4. Differentially abundant human- and bovine-derived proteins in feces during the first six postnatal weeks between gestational age groups preterm (25 – 31 weeks of gestation) and term (≥37 weeks of gestation).

Table S5. Human- and bovine-derived proteins identified in more than 50% of the gastric and fecal proteomes of preterm infants (25 – 31 weeks of gestation).

Table S6. Bacterial oxidative stress proteins from opportunistic pathogens including *Enterococcus* spp., *Escherichia* spp. and *Klebsiella* spp.

Figure S1. Cumulative relative abundance of casein fragments in feces of preterm and term infants during the first six postnatal weeks.

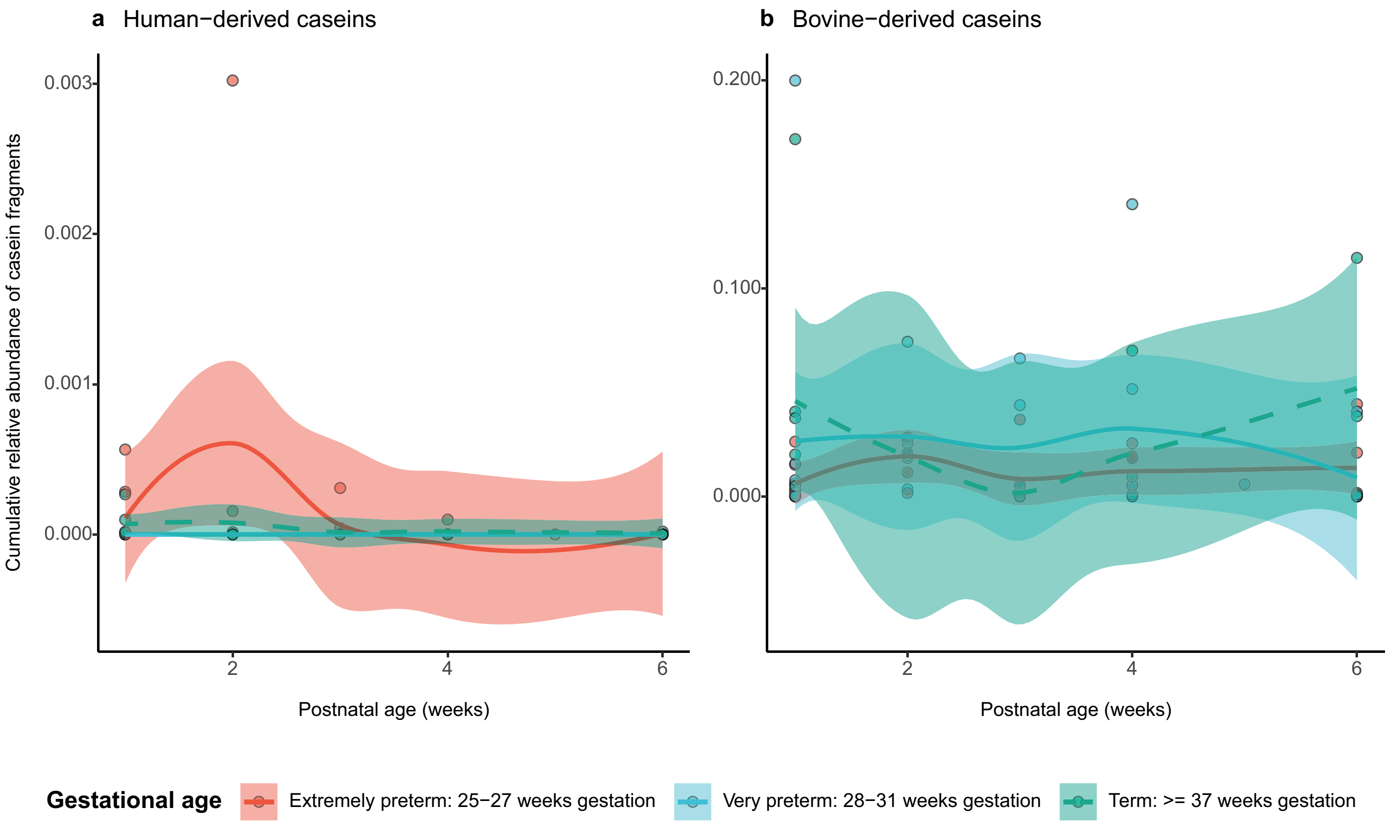

(a) Human-derived and (b) bovine-derived alpha-, beta- and kappa-casein fragments in feces of preterm and term infants during the first six postnatal weeks. riBAQ was used to calculate relative abundances and were calculated with respect to all human-derived or all bovine-derived proteins. Non-parametric LOESS regression with a 95% confidence interval was used to generate a smooth fitted line per gestational age group.

Figure S2. Distribution of proteins derived from human, bovine, human or bovine and bacterial source per postnatal week.

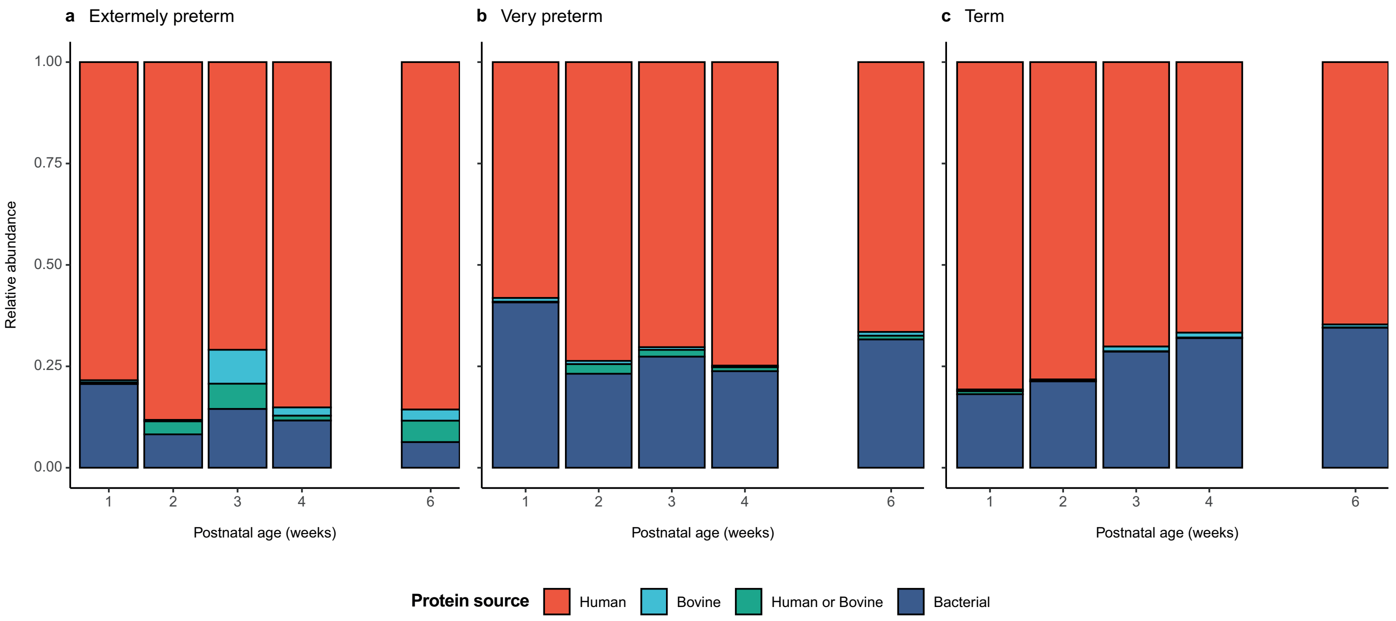

Distribution of proteins identified in feces of (a) extremely preterm, (b) very preterm and (c) term infants. riBAQ was applied by dividing by the sum of all proteins.

Figure S3. Distribution of proteins derived from bacterial genera *Bifidobacterium*, *Enterococcus*, *Klebsiella* and other genera per postnatal week.

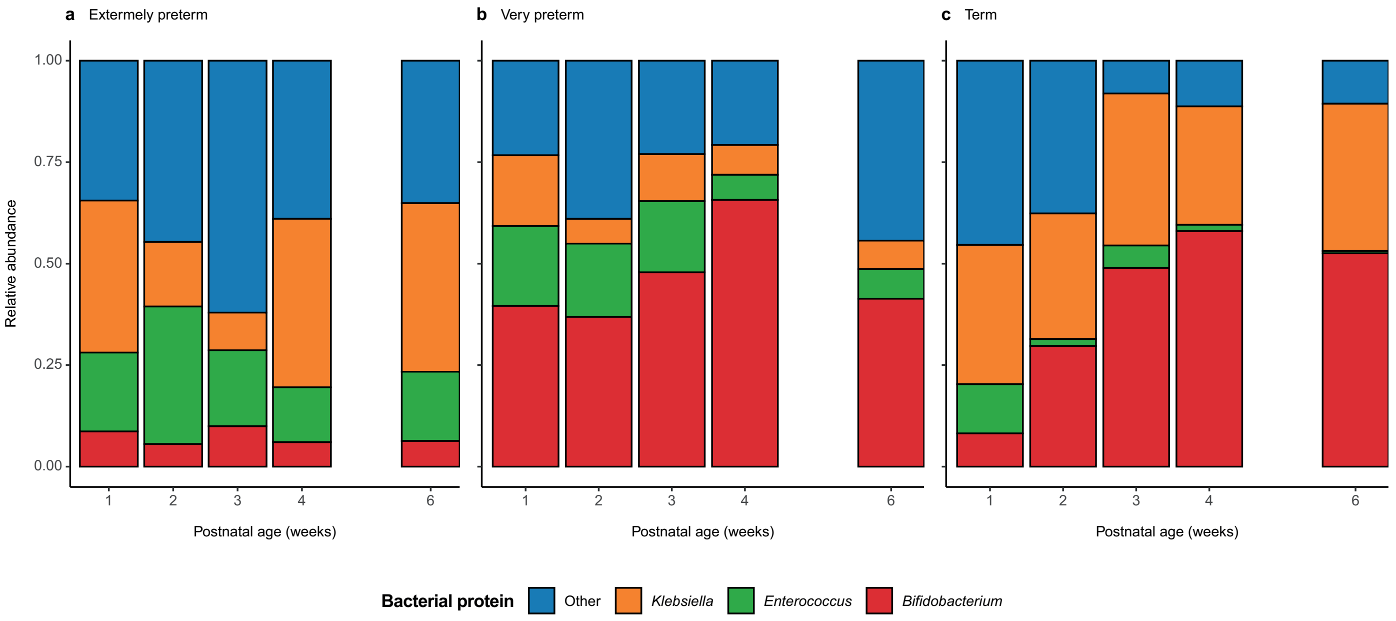

Distribution of proteins identified in feces of (a) extremely preterm, (b) very preterm and (c) term infants. riBAQ was applied by dividing by the sum of all bacterial proteins.

Figure S4. Spearman correlations between preterm infant's relative abundance of *Bifidobacterium*-derived proteins in postnatal weeks 1-6 and birth weight.

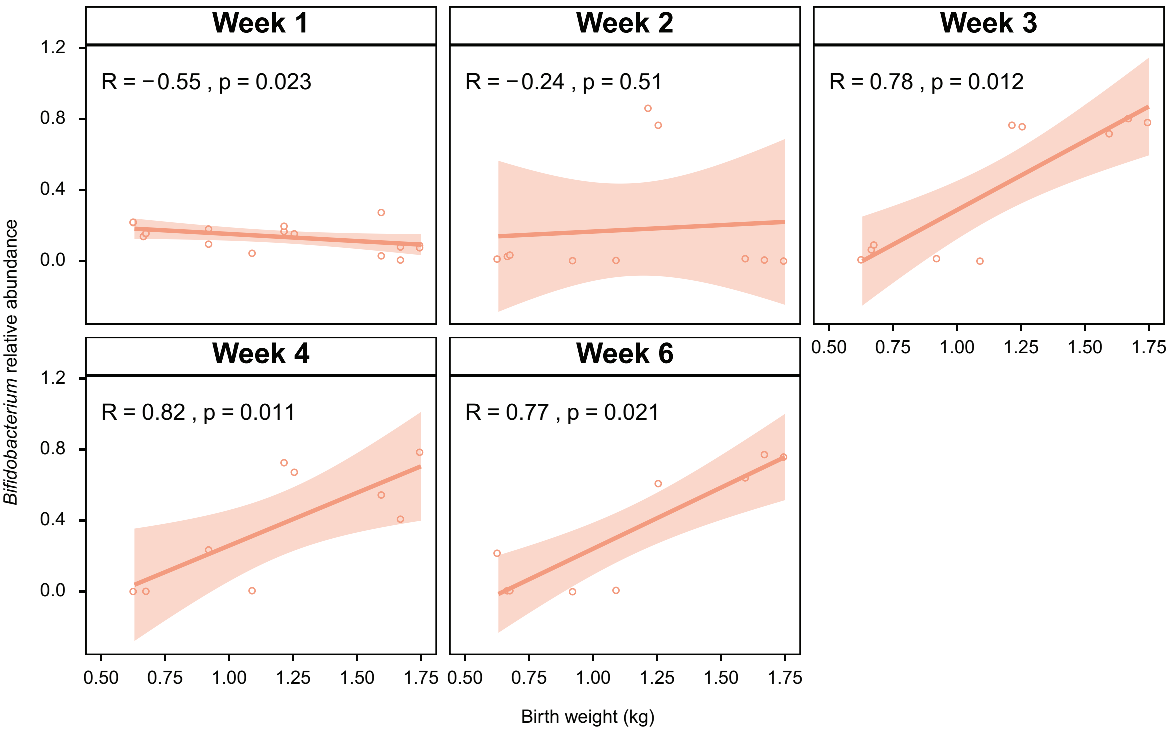

Bifidobacterium-derived proteins as displayed on the y-axis were calculated using riBAQ with respect to all bacterial-derived proteins. *Bifidobacterium* relative abundance is displayed in the panel's corresponding postnatal week. Birth weight in kilograms is displayed on the x-axis.

Figure S5. Spearman correlations between preterm infant's relative abundance of *Bifidobacterium*-derived proteins in postnatal weeks 1-6 and growth velocity.

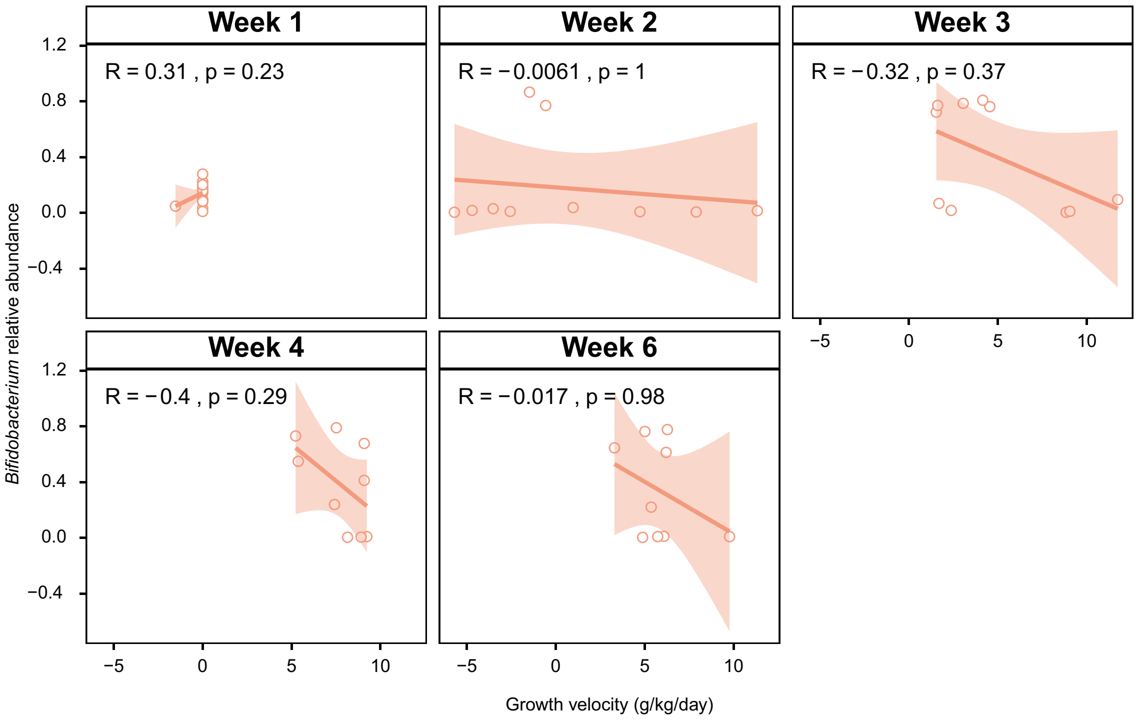

Bifidobacterium-derived proteins as displayed on the y-axis were calculated using riBAQ with respect to all bacterial-derived proteins. *Bifidobacterium* relative abundance are displayed in the panel's corresponding postnatal week. Growth velocity in g/kg/day is displayed on the x-axis.

Figure S6. Relative abundance of bacterial oxidative stress proteins per postnatal week.

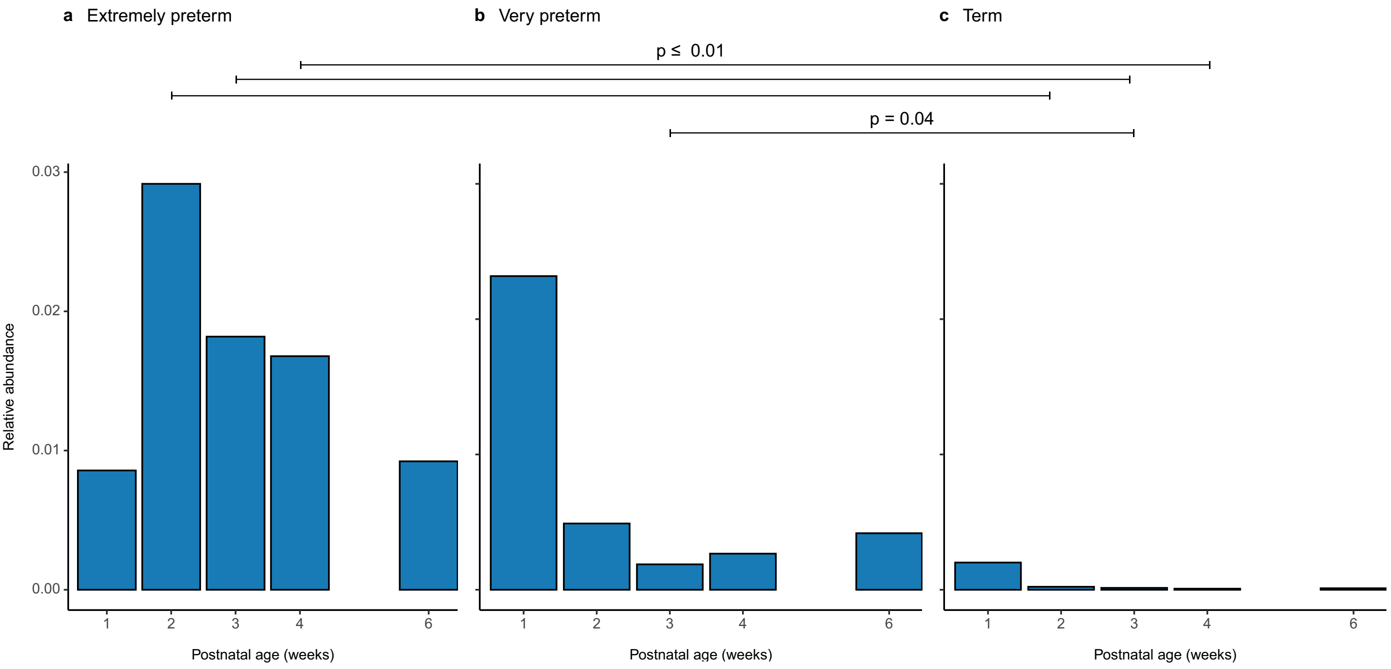

Bacterial oxidative stress proteins from opportunistic pathogens including *Enterococcus* spp., *Escherichia* spp. and *Klebsiella* spp. identified in feces of (a) extremely preterm, (b) very preterm and (c) term infants. riBAQ was applied by dividing by the sum of all bacterial proteins.

Figure S7. Principal component analysis on the gastric proteome of preterm infants during the first two postnatal weeks.

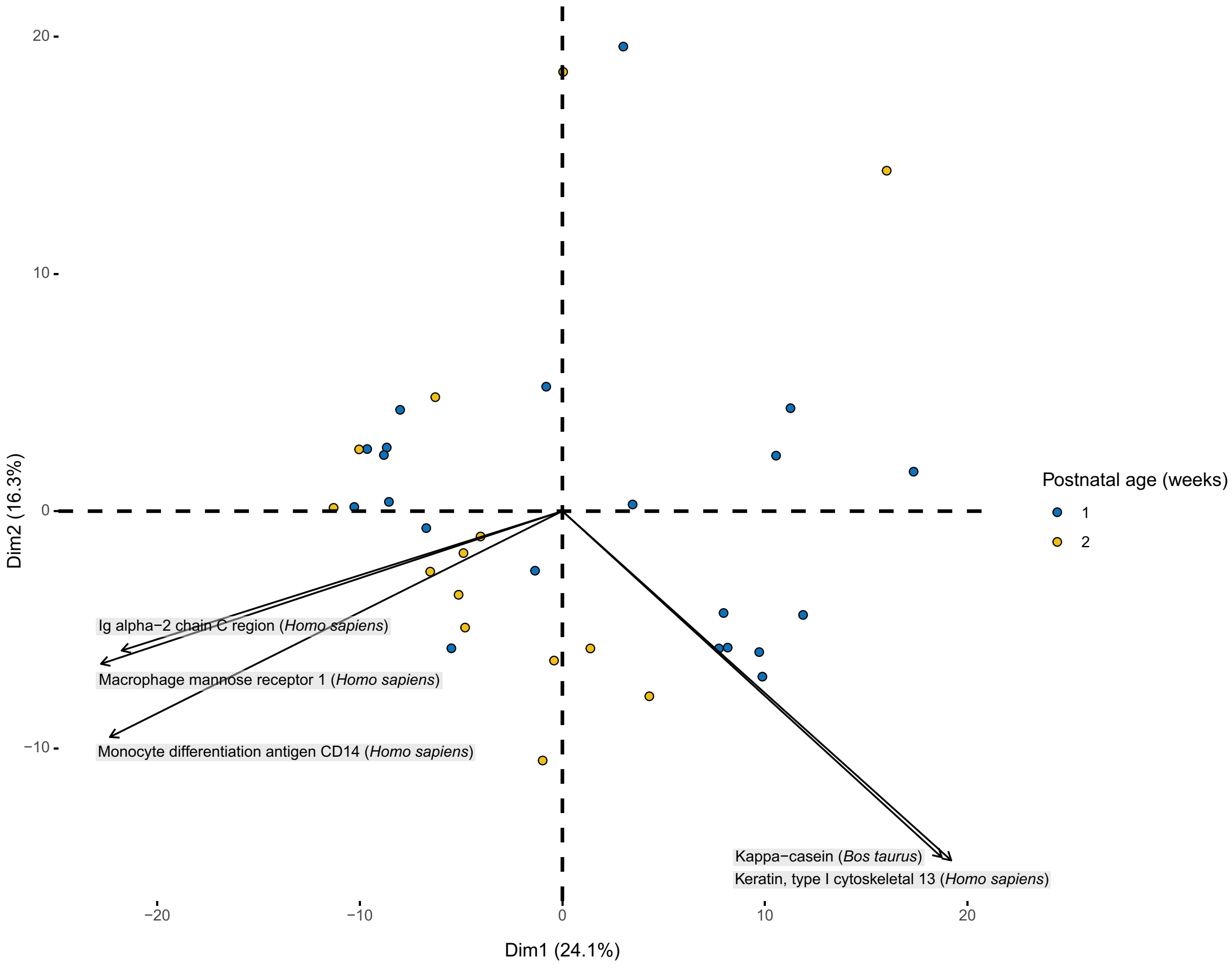

Arrows display proteins explaining most variation on the first two principal components. Colored points indicate infant samples of one timepoint.

Table S1. Infant characteristics of two subsets of the EIBER cohort.

a.

	Gestational age (mean ± SD)	Birth weight (grams)	Female (%)	C-section (%)	Food Intolerance (%)[a]	Enteral feeding (%)[b, c]	Human milk (%)[b, c, d]
ALL (n = 40)	28.5 ± 2.4	1177 ± 393	47.5	57.5	22.5	39, 88	72, 95
EP (n = 14)	25.9 ± 1.2	804 ± 156	42.9	50.0	35.7	28, 77	74, 91
VP (n = 23)	29.5 ± 1.0	1327 ± 294	52.2	56.5	17.4	47, 95	71, 97
MLP (n = 3)	33.0 ± 0.0	1768 ± 382	33.3	100.0	0.0	-	-

b.

	Gestational age (mean ± SD)	Birth weight (kg)	Weight gain (kg)	Female (%)	C-section (%)	Food Intolerance (%)[a]	Enteral feeding (%)[e]	Human milk (%)[d, e]
EP (n = 5)	26.2 ± 0.8	0.80 ± 0.18	0.74 ± 0.16	20.0	80.0	80.0	22, 66, 79, 87, 100	20, 66, 68, 86, 77
VP (n = 5)	30.0 ± 0.0	1.52 ± 0.22	0.71 ± 0.15	80.0	80.0	20.0	42, 89, 100, 100, 100	18, 84, 91, 82, 65
T (n = 3)	38.7 ± 2.1	3.22 ± 0.68	-	33.3	66.7	0.0	100,100,100,100,100	50, 98, 69, 67,67

Infant characteristics of two subsets of the EIBER cohort: (a) Forty preterm infants of whom pH, protease and pepsin activity were analyzed daily during the first two postnatal weeks; and (b) Ten preterm infants of whom gastric proteome was analyzed weekly in the first two weeks and fecal proteome was analyzed weekly during the first six postnatal weeks, as well as three term infants of whom fecal proteome was analyzed weekly during the first six postnatal weeks. EP: extremely preterm, VP: very preterm, MLP: moderate to late preterm, T: term. [a] Defined as infants not bearing an increase in amount of food as assessed by (increasing) retention, vomiting or diarrhea or the appearance of clinical symptoms including tense and extended abdomen, diminished bowel movements or bloody stool. [b] Data was available for 35 infants; 14 EP and 21 VP infants. For one out of three MLP infants, data was available and was therefore not included in analyses. [c] Percentage at postnatal weeks 1 and 2 respectively. [d] Percentage of human milk as enteral feeding. [e] Percentage at postnatal weeks 1, 2, 3, 4 and 6 respectively.

Table S2. Differentially abundant human- and bovine-derived proteins between gastric and fecal samples of preterm infants during postnatal weeks one and two.

a.

**Fasta header**	**Gene name**	**Protein name**	**-Log Student's T-test p-value**	**Student's T-test q-value**	**Student's T-test Difference**	**Student's T-test Test statistic**
>sp|P00709|LALBA_HUMAN Alpha-lactalbumin OS=Homo sapiens GN=LALBA PE=1 SV=1; >tr|F8VWU1|F8VWU1_HUMAN Alpha-lactalbumin OS=Homo sapiens GN=LALBA PE=3 SV=1	LALBA	Alpha-lactalbumin	21.44	0.00	-4.50	-3.47
>sp|P07498|CASK_HUMAN Kappa-casein OS=Homo sapiens GN=CSN3 PE=1 SV=3	CSN3	Kappa-casein	25.18	0.00	-4.37	-3.51
>sp|P05814|CASB_HUMAN Beta-casein OS=Homo sapiens GN=CSN2 PE=1 SV=4	CSN2	Beta-casein	17.14	0.00	-4.21	-3.13
>sp|P10909|CLUS_HUMAN Clusterin OS=Homo sapiens GN=CLU PE=1 SV=1; >tr|E7ERK6|E7ERK6_HUMAN Clusterin beta chain (Fragment) OS=Homo sapiens GN=CLU PE=4 SV=1	CLU	Clusterin; Clusterin beta chain	22.13	0.00	-3.95	-3.15
>sp|P47710|CASA1_HUMAN Alpha-S1-casein OS=Homo sapiens GN=CSN1S1 PE=1 SV=1; >tr|E9PDQ1|E9PDQ1_HUMAN Alpha-S1-casein OS=Homo sapiens GN=CSN1S1 PE=1 SV=1	CSN1S1	Alpha-S1-casein; Casoxin-D	16.86	0.00	-3.88	-2.93
>sp|P03973|SLPI_HUMAN Antileukoproteinase OS=Homo sapiens GN=SLPI PE=1 SV=2	SLPI	Antileukoproteinase	23.68	0.00	-3.54	-2.93
>sp|P12273|PIP_HUMAN Prolactin-inducible protein OS=Homo sapiens GN=PIP PE=1 SV=1	PIP	Prolactin-inducible protein	17.37	0.00	-3.54	-2.75
>sp|Q13410|BT1A1_HUMAN Butyrophilin subfamily 1 member A1 OS=Homo sapiens GN=BTN1A1 PE=1 SV=3; >tr|Q4VAN1|Q4VAN1_HUMAN BTN1A1 protein OS=Homo sapiens GN=BTN1A1 PE=2 SV=1	BTN1A1	Butyrophilin subfamily 1 member A1	12.27	0.00	-3.16	-2.35
>sp|Q6WN34|CRDL2_HUMAN Chordin-like protein 2 OS=Homo sapiens GN=CHRDL2 PE=1 SV=1; >tr|A0A087WZH6|A0A087WZH6_HUMAN Chordin-like protein 2 OS=Homo sapiens GN=CHRDL2 PE=4 SV=1	CHRDL2; CHL2	Chordin-like protein 2	14.36	0.00	-3.13	-2.41
>tr|G3V2V8|G3V2V8_HUMAN Epididymal secretory protein E1 (Fragment) OS=Homo sapiens GN=NPC2 PE=1 SV=1; >tr|J3KMY5|J3KMY5_HUMAN Epididymal secretory protein E1 OS=Homo sapiens GN=NPC2 PE=1 SV=1	NPC2	Epididymal secretory protein E1	16.92	0.00	-3.08	-2.45
>sp|P06727|APOA4_HUMAN Apolipoprotein A-IV OS=Homo sapiens GN=APOA4 PE=1 SV=3	APOA4	Apolipoprotein A-IV	25.56	0.00	-3.05	-2.62

Fasta header	Gene name	Protein name	-Log Student's T-test p-value	Student's T-test q-value	Student's T-test Difference	Student's T-test Test statistic
>sp|P02765|FETUA_HUMAN Alpha-2-HS-glycoprotein OS=Homo sapiens GN=AHSG PE=1 SV=1; >tr|C9JV77|C9JV77_HUMAN Alpha-2-HS-glycoprotein OS=Homo sapiens GN=AHSG PE=1 SV=1	AHSG	Alpha-2-HS-glycoprotein; Alpha-2-HS-glycoprotein chain A	18.59	0.00	-3.03	-2.46
>sp|P18065|IBP2_HUMAN Insulin-like growth factor-binding protein 2 OS=Homo sapiens GN=IGFBP2 PE=1 SV=2; >tr|C9JMY1|C9JMY1_HUMAN Insulin-like growth factor-binding protein 2 OS=Homo sapiens GN=IGFBP2 PE=4 SV=1	IGFBP 2	Insulin-like growth factor-binding protein 2	19.27	0.00	-2.92	-2.41
>sp|P02774|VTDB_HUMAN Vitamin D-binding protein OS=Homo sapiens GN=GC PE=1 SV=1; >tr|D6RBJ7|D6RBJ7_HUMAN Vitamin D-binding protein OS=Homo sapiens GN=GC PE=1 SV=1	GC	Vitamin D-binding protein	14.83	0.00	-2.91	-2.29
>sp|P61626|LYSC_HUMAN Lysozyme C OS=Homo sapiens GN=LYZ PE=1 SV=1; >tr|F8VV32|F8VV32_HUMAN Lysozyme C OS=Homo sapiens GN=LYZ PE=1 SV=1	LYZ	Lysozyme C	12.84	0.00	-2.90	-2.22
>tr|B4E1Z4|B4E1Z4_HUMAN Uncharacterized protein OS=Homo sapiens PE=2 SV=1; >sp|P00751|CFAB_HUMAN Complement factor B OS=Homo sapiens GN=CFB PE=1 SV=2	CFB	Complement factor B; Complement factor B Ba fragment	16.48	0.00	-2.84	-2.29
>sp|P19013|K2C4_HUMAN Keratin, type II cytoskeletal 4 OS=Homo sapiens GN=KRT4 PE=1 SV=4; >sp|P19013|K2C4_HUMAN Keratin, type II cytoskeletal 4 OS=Homo sapiens GN=KRT4 PE=1 SV=4	KRT4	Keratin, type II cytoskeletal 4	9.50	0.00	-2.79	-2.04
>sp|Q08431|MFGM_HUMAN Lactadherin OS=Homo sapiens GN=MFGE8 PE=1 SV=2; >tr|F5GZN3|F5GZN3_HUMAN Lactadherin short form OS=Homo sapiens GN=MFGE8 PE=4 SV=1	MFGE8	Lactadherin; Lactadherin short form	9.03	0.00	-2.78	-2.01
>sp|P02749|APOH_HUMAN Beta-2-glycoprotein 1 OS=Homo sapiens GN=APOH PE=1 SV=3; >tr|J3QLI0|J3QLI0_HUMAN Beta-2-glycoprotein 1 (Fragment) OS=Homo sapiens GN=APOH PE=4 SV=1	APOH	Beta-2-glycoprotein 1	11.56	0.00	-2.77	-2.10
>sp|P24821|TENA_HUMAN Tenascin OS=Homo sapiens GN=TNC PE=1 SV=3;>tr|J3QSU6|J3QSU6_HUMAN Tenascin OS=Homo sapiens GN=TNC PE=1 SV=1; >tr|E9PC84|E9PC84_HUMAN Tenascin OS=Homo sapiens GN=TNC PE=1 SV=1	TNC	Tenascin	8.39	0.00	-2.77	-1.98
>sp|P01833|PIGR_HUMAN Polymeric immunoglobulin receptor OS=Homo sapiens GN=PIGR PE=1 SV=4	PIGR	Polymeric immunoglobulin receptor; Secretory component	11.71	0.00	-2.75	-2.10

Fasta header	Gene name	Protein name	-Log Student's T-test p-value	Student's T-test q-value	Student's T-test Difference	Student's T-test Test statistic
>sp|Q8TDL5|BPIB1_HUMAN BPI fold-containing family B member 1 OS=Homo sapiens GN=BPIFB1 PE=1 SV=1	BPIFB1	BPI fold-containing family B member 1	9.28	0.00	-2.73	-1.99
>sp|Q0P569|NUCB1_BOVIN Nucleobindin-1 OS=Bos taurus GN=NUCB1 PE=2 SV=1; >sp|Q02818|NUCB1_HUMAN Nucleobindin-1 OS=Homo sapiens GN=NUCB1 PE=1 SV=4	NUCB1	Nucleobindin-1	16.26	0.00	-2.70	-2.19
>tr|J3KPS3|J3KPS3_HUMAN Fructose-bisphosphate aldolase OS=Homo sapiens GN=ALDOA PE=1 SV=1; >sp|P04075|ALDOA_HUMAN Fructose-bisphosphate aldolase A OS=Homo sapiens GN=ALDOA PE=1 SV=2	ALDOA	Fructose-bisphosphate aldolase A	10.93	0.00	-2.67	-2.02
>sp|P00738|HPT_HUMAN Haptoglobin OS=Homo sapiens GN=HP PE=1 SV=1; >tr|J3QLC9|J3QLC9_HUMAN Haptoglobin (Fragment) OS=Homo sapiens GN=HP PE=1 SV=1	HP	Haptoglobin	12.97	0.00	-2.65	-2.08
>sp|P08603|CFAH_HUMAN Complement factor H OS=Homo sapiens GN=CFH PE=1 SV=4	CFH	Complement factor H	12.92	0.00	-2.58	-2.03
>sp|P02649|APOE_HUMAN Apolipoprotein E OS=Homo sapiens GN=APOE PE=1 SV=1; >tr|E9PEV4|E9PEV4_HUMAN Apolipoprotein E (Fragment) OS=Homo sapiens GN=APOE PE=1 SV=1	APOE	Apolipoprotein E	10.85	0.00	-2.54	-1.95
>tr|X6R8F3|X6R8F3_HUMAN Neutrophil gelatinase-associated lipocalin OS=Homo sapiens GN=LCN2 PE=1 SV=1; >sp|P80188|NGAL_HUMAN Neutrophil gelatinase-associated lipocalin OS=Homo sapiens GN=LCN2 PE=1 SV=2	LCN2	Neutrophil gelatinase-associated lipocalin	12.70	0.00	-2.52	-1.99
>sp|P02671|FIBA_HUMAN Fibrinogen alpha chain OS=Homo sapiens GN=FGA PE=1 SV=2; >tr|A0A087WUA0|A0A087WUA0_HUMAN Fibrinogen alpha chain OS=Homo sapiens GN=FGA PE=1 SV=1	FGA	Fibrinogen alpha chain	10.63	0.00	-2.50	-1.92
>tr|Q2HJ20|Q2HJ20_BOVIN Sclerostin domain containing 1 OS=Bos taurus GN=SOSTDC1 PE=2 SV=1; >sp|Q6X4U4|SOSD1_HUMAN Sclerostin domain-containing protein 1 OS=Homo sapiens GN=SOSTDC1 PE=1 SV=2	SOSTDC1	Sclerostin domain-containing protein 1	8.31	0.00	-2.47	-1.82
>sp|Q9UBC9|SPRR3_HUMAN Small proline-rich protein 3 OS=Homo sapiens GN=SPRR3 PE=1 SV=2; >tr|B1AN48|B1AN48_HUMAN Small proline-rich protein 3 (Fragment) OS=Homo sapiens GN=SPRR3 PE=4 SV=3	SPRR3	Small proline-rich protein 3	12.02	0.00	-2.40	-1.90
>tr|A0A087WYF5|A0A087WYF5_HUMAN Salivary acidic proline-rich phosphoprotein 1/2 (Fragment) OS=Homo sapiens GN=PRH1 PE=4 SV=1;	PRH1	Salivary acidic proline-rich phosphoprotein 1/2	11.99	0.00	-2.40	-1.89

Fasta header	Gene name	Protein name	-Log Student's T-test p-value	Student's T-test q-value	Student's T-test Difference	Student's T-test Test statistic
>tr|A0A087WYT0|A0A087WYT0_HUMAN Salivary acidic proline-rich phosphoprotein 1/2 OS=Homo sapiens GN=PRH1 PE=4 SV=1						
>sp|Q9HC84|MUC5B_HUMAN Mucin-5B OS=Homo sapiens GN=MUC5B PE=1 SV=3	MUC5B	Mucin-5B	8.77	0.00	-2.32	-1.75
>tr|A2VE41|A2VE41_BOVIN EGF-containing fibulin-like extracellular matrix protein 1 OS=Bos taurus GN=EFEMP1 PE=2 SV=1;<br>>sp|Q12805|FBLN3_HUMAN EGF-containing fibulin-like extracellular matrix protein 1 OS=Homo sapiens GN=EFEMP1 PE=1 SV=2	EFEMP1	EGF-containing fibulin-like extracellular matrix protein 1	12.16	0.00	-2.28	-1.82
>sp|P02647|APOA1_HUMAN Apolipoprotein A-I OS=Homo sapiens GN=APOA1 PE=1 SV=1;<br>>tr|F8W696|F8W696_HUMAN Truncated apolipoprotein A-I OS=Homo sapiens GN=APOA1 PE=1 SV=1	APOA1	Apolipoprotein A-I;<br>Proapolipoprotein A-I	9.93	0.00	-2.27	-1.76
>sp|P08571|CD14_HUMAN Monocyte differentiation antigen CD14 OS=Homo sapiens GN=CD14 PE=1 SV=2;<br>>tr|D6RFL4|D6RFL4_HUMAN Monocyte differentiation antigen CD14, urinary form (Fragment) OS=Homo sapiens GN=CD14 PE=4 SV=1	CD14	Monocyte differentiation antigen CD14;<br>Monocyte differentiation antigen CD14, urinary form	5.71	0.00	-2.25	-1.58
>tr|X6R868|X6R868_HUMAN Bile salt-activated lipase OS=Homo sapiens GN=CEL PE=3 SV=1;<br>>sp|P19835|CEL_HUMAN Bile salt-activated lipase OS=Homo sapiens GN=CEL PE=1 SV=3	CEL	Bile salt-activated lipase	6.05	0.00	-2.21	-1.57
>sp|P0C0L4|CO4A_HUMAN Complement C4-A OS=Homo sapiens GN=C4A PE=1 SV=2;<br>>sp|P0C0L5|CO4B_HUMAN Complement C4-B OS=Homo sapiens GN=C4B PE=1 SV=2	C4A;<br>C4B	Complement C4-A;<br>Complement C4 beta chain	7.39	0.00	-2.20	-1.63
>sp|P07098|LIPG_HUMAN Gastric triacylglycerol lipase OS=Homo sapiens GN=LIPF PE=1 SV=1	LIPF	Gastric triacylglycerol lipase	7.59	0.00	-2.18	-1.63
>sp|P59666|DEF3_HUMAN Neutrophil defensin 3 OS=Homo sapiens GN=DEFA3 PE=1 SV=1;<br>>sp|P59665|DEF1_HUMAN Neutrophil defensin 1 OS=Homo sapiens GN=DEFA1 PE=1 SV=1	DEFA3;<br>DEFA1	Neutrophil defensin 3;HP 3-56;<br>Neutrophil defensin 1;HP 1-56	6.74	0.00	-2.17	-1.59
>sp|Q03403|TFF2_HUMAN Trefoil factor 2 OS=Homo sapiens GN=TFF2 PE=1 SV=2	TFF2	Trefoil factor 2	11.48	0.00	-2.11	-1.70
>sp|O00391|QSOX1_HUMAN Sulfhydryl oxidase 1 OS=Homo sapiens GN=QSOX1 PE=1 SV=3	QSOX1	Sulfhydryl oxidase 1	5.57	0.00	-2.10	-1.50
>tr|A0A087WXL8|A0A087WXL8_HUMAN Ig gamma-3 chain C region OS=Homo sapiens GN=IGHG3 PE=4 SV=1;	IGHG3	Ig gamma-3 chain C region	5.72	0.00	-2.03	-1.47

Fasta header	Gene name	Protein name	-Log Student's T-test p-value	Student's T-test q-value	Student's T-test Difference	Student's T-test Test statistic
>tr|A0A075B6N8|A0A075B6N8_HUMAN Ig gamma-3 chain C region (Fragment) OS=Homo sapiens GN=IGHG3 PE=4 SV=1						
>sp|P43652|AFAM_HUMAN Afamin OS=Homo sapiens GN=AFM PE=1 SV=1	AFM	Afamin	9.85	0.00	-2.02	-1.60
>sp|P10451|OSTP_HUMAN Osteopontin OS=Homo sapiens GN=SPP1 PE=1 SV=1; >tr|D6R9C5|D6R9C5_HUMAN Osteopontin (Fragment) OS=Homo sapiens GN=SPP1 PE=1 SV=1	SPP1	Osteopontin	5.57	0.00	-1.98	-1.44
>sp|Q9P2E9|RRBP1_HUMAN Ribosome-binding protein 1 OS=Homo sapiens GN=RRBP1 PE=1 SV=4; >tr|A0A087WVV2|A0A087WVV2_HUMAN Ribosome-binding protein 1 OS=Homo sapiens GN=RRBP1 PE=1 SV=1	RRBP1	Ribosome-binding protein 1	8.94	0.00	-1.98	-1.55
>tr|C9JIZ6|C9JIZ6_HUMAN Prosaposin OS=Homo sapiens GN=PSAP PE=1 SV=2; >tr|B1AVU8|B1AVU8_HUMAN Saposin-D OS=Homo sapiens GN=PSAP PE=1 SV=1	PSAP	Prosaposin; Saposin-D	7.78	0.00	-1.97	-1.51
>sp|P22897|MRC1_HUMAN Macrophage mannose receptor 1 OS=Homo sapiens GN=MRC1 PE=1 SV=1	MRC1	Macrophage mannose receptor 1	5.69	0.00	-1.93	-1.41
>sp|P13646|K1C13_HUMAN Keratin, type I cytoskeletal 13 OS=Homo sapiens GN=KRT13 PE=1 SV=4; >tr|K7ERE3|K7ERE3_HUMAN Keratin, type I cytoskeletal 13 OS=Homo sapiens GN=KRT13 PE=1 SV=1	KRT13	Keratin, type I cytoskeletal 13	5.54	0.00	-1.93	-1.40
>tr|S4R371|S4R371_HUMAN Fatty acid-binding protein, heart (Fragment) OS=Homo sapiens GN=FABP3 PE=1 SV=1; >sp|P05413|FABPH_HUMAN Fatty acid-binding protein, heart OS=Homo sapiens GN=FABP3 PE=1 SV=4	FABP3	Fatty acid-binding protein, heart	4.91	0.00	-1.86	-1.34
>sp|P16403|H12_HUMAN Histone H1.2 OS=Homo sapiens GN=HIST1H1C PE=1 SV=2; >sp|A7MAZ5|H13_BOVIN Histone H1.3 OS=Bos taurus GN=HIST1H1D PE=1 SV=1	HIST1 H1C; HIST1 H1D	Histone H1.2	5.86	0.00	-1.86	-1.38
>sp|P05362|ICAM1_HUMAN Intercellular adhesion molecule 1 OS=Homo sapiens GN=ICAM1 PE=1 SV=2; >tr|E7ESS4|E7ESS4_HUMAN Intercellular adhesion molecule 1 OS=Homo sapiens GN=ICAM1 PE=1 SV=1	ICAM1	Intercellular adhesion molecule 1	9.14	0.00	-1.84	-1.47
>tr|E7EU05|E7EU05_HUMAN Platelet glycoprotein 4 (Fragment) OS=Homo sapiens GN=CD36 PE=4 SV=2; >sp|P16671|CD36_HUMAN Platelet glycoprotein 4 OS=Homo sapiens GN=CD36 PE=1 SV=2	CD36	Platelet glycoprotein 4	4.65	0.00	-1.83	-1.31

Fasta header	Gene name	Protein name	-Log Student's T-test p-value	Student's T-test q-value	Student's T-test Difference	Student's T-test Test statistic
>sp|P02787|TRFE_HUMAN Serotransferrin OS=Homo sapiens GN=TF PE=1 SV=3	TF	Serotransferrin	6.68	0.00	-1.83	-1.40
>sp|P36222|CH3L1_HUMAN Chitinase-3-like protein 1 OS=Homo sapiens GN=CHI3L1 PE=1 SV=2; >tr|H0Y3U8|H0Y3U8_HUMAN Chitinase-3-like protein 1 (Fragment) OS=Homo sapiens GN=CHI3L1 PE=3 SV=1	CHI3L1	Chitinase-3-like protein 1	5.53	0.00	-1.83	-1.35
>sp|P35321|SPR1A_HUMAN Cornifin-A OS=Homo sapiens GN=SPRR1A PE=1 SV=2	SPRR1 A	Cornifin-A	6.18	0.00	-1.76	-1.34
>sp|P02538|K2C6A_HUMAN Keratin, type II cytoskeletal 6A OS=Homo sapiens GN=KRT6A PE=1 SV=3	KRT6A	Keratin, type II cytoskeletal 6A	4.11	0.00	-1.75	-1.24
>sp|Q99541|PLIN2_HUMAN Perilipin-2 OS=Homo sapiens GN=PLIN2 PE=1 SV=2	PLIN2	Perilipin-2	5.13	0.00	-1.71	-1.27
>sp|P06733|ENOA_HUMAN Alpha-enolase OS=Homo sapiens GN=ENO1 PE=1 SV=2	ENO1	Alpha-enolase	5.58	0.00	-1.68	-1.27
>sp|P07996|TSP1_HUMAN Thrombospondin-1 OS=Homo sapiens GN=THBS1 PE=1 SV=2; >sp|Q28178|TSP1_BOVIN Thrombospondin-1 OS=Bos taurus GN=THBS1 PE=2 SV=2	THBS1	Thrombospondin-1	4.01	0.00	-1.61	-1.16
>tr|H0YL18|H0YL18_HUMAN Beta-2-microglobulin form pI 5.3 OS=Homo sapiens GN=B2M PE=1 SV=1; >sp|P61769|B2MG_HUMAN Beta-2-microglobulin OS=Homo sapiens GN=B2M PE=1 SV=1	B2M	Beta-2-microglobulin; Beta-2-microglobulin form pI 5.3	4.75	0.00	-1.60	-1.19
>sp|P06396|GELS_HUMAN Gelsolin OS=Homo sapiens GN=GSN PE=1 SV=1;>tr|Q5T0H9|Q5T0H9_HUMAN Gelsolin OS=Homo sapiens GN=GSN PE=1 SV=1; >tr|Q5T0H8|Q5T0H8_HUMAN Gelsolin OS=Homo sapiens GN=GSN PE=1 SV=1	GSN	Gelsolin	4.23	0.00	-1.59	-1.16
>sp|P36871|PGM1_HUMAN Phosphoglucomutase-1 OS=Homo sapiens GN=PGM1 PE=1 SV=3; >sp|Q08DP0|PGM1_BOVIN Phosphoglucomutase-1 OS=Bos taurus GN=PGM1 PE=2 SV=1	PGM1	Phosphoglucomutase-1	5.13	0.00	-1.51	-1.16
>sp|P16401|H15_HUMAN Histone H1.5 OS=Homo sapiens GN=HIST1H1B PE=1 SV=3	HIST1 H1B	Histone H1.5	3.47	0.00	-1.50	-1.07
>sp|P04217|A1BG_HUMAN Alpha-1B-glycoprotein OS=Homo sapiens GN=A1BG PE=1 SV=4; >tr|M0R009|M0R009_HUMAN Alpha-1B-glycoprotein (Fragment) OS=Homo sapiens GN=A1BG PE=4 SV=3	A1BG	Alpha-1B-glycoprotein	4.58	0.00	-1.49	-1.13
>sp|P05204|HMGN2_HUMAN Non-histone chromosomal protein HMG-17 OS=Homo sapiens GN=HMGN2 PE=1 SV=3	HMGN 2	Non-histone chromosomal protein HMG-17	6.23	0.00	-1.49	-1.17

Fasta header	Gene name	Protein name	-Log Student's T-test p-value	Student's T-test q-value	Student's T-test Difference	Student's T-test Test statistic
>sp|P02771|FETA_HUMAN Alpha-fetoprotein OS=Homo sapiens GN=AFP PE=1 SV=1; >tr|J3KMX3|J3KMX3_HUMAN Alpha-fetoprotein OS=Homo sapiens GN=AFP PE=1 SV=1	AFP	Alpha-fetoprotein	3.14	0.00	-1.47	-1.04
>sp|O95171|SCEL_HUMAN Sciellin OS=Homo sapiens GN=SCEL PE=1 SV=2	SCEL	Sciellin	4.36	0.00	-1.46	-1.10
>tr|E7ENC5|E7ENC5_HUMAN Mucin-4 beta chain OS=Homo sapiens GN=MUC4 PE=4 SV=1; >tr|E9PDY6|E9PDY6_HUMAN Mucin-4 beta chain OS=Homo sapiens GN=MUC4 PE=4 SV=1	MUC4	Mucin-4 beta chain	4.36	0.00	-1.45	-1.09
>tr|A0A087WZH7|A0A087WZH7_HUMAN Myristoylated alanine-rich C-kinase substrate OS=Homo sapiens GN=MARCKS PE=1 SV=1; >sp|P29966|MARCS_HUMAN Myristoylated alanine-rich C-kinase substrate OS=Homo sapiens GN=MARCKS PE=1 SV=4	MARCKS	Myristoylated alanine-rich C-kinase substrate	6.14	0.00	-1.44	-1.14
>tr|E9PNW4|E9PNW4_HUMAN CD59 glycoprotein OS=Homo sapiens GN=CD59 PE=1 SV=1; >tr|E9PR17|E9PR17_HUMAN CD59 glycoprotein OS=Homo sapiens GN=CD59 PE=1 SV=1	CD59	CD59 glycoprotein	3.99	0.00	-1.41	-1.05
>sp|P02768|ALBU_HUMAN Serum albumin OS=Homo sapiens GN=ALB PE=1 SV=2; >tr|B7WNR0|B7WNR0_HUMAN Serum albumin OS=Homo sapiens GN=ALB PE=1 SV=1	ALB	Serum albumin	8.71	0.00	-1.40	-1.17
>sp|P69892|HBG2_HUMAN Hemoglobin subunit gamma-2 OS=Homo sapiens GN=HBG2 PE=1 SV=2; >tr|E9PBW4|E9PBW4_HUMAN Hemoglobin subunit gamma-2 OS=Homo sapiens GN=HBG2 PE=1 SV=1	HBG2	Hemoglobin subunit gamma-2	3.28	0.00	-1.37	-1.00
>sp|P25311|ZA2G_HUMAN Zinc-alpha-2-glycoprotein OS=Homo sapiens GN=AZGP1 PE=1 SV=2; >tr|C9JEV0|C9JEV0_HUMAN Zinc-alpha-2-glycoprotein OS=Homo sapiens GN=AZGP1 PE=3 SV=1	AZGP1	Zinc-alpha-2-glycoprotein	4.23	0.00	-1.37	-1.04
>tr|A0A087X2C0|A0A087X2C0_HUMAN Ig mu chain C region OS=Homo sapiens GN=IGHM PE=1 SV=1; >tr|A0A075B6N9|A0A075B6N9_HUMAN Ig mu chain C region (Fragment) OS=Homo sapiens GN=IGHM PE=1 SV=2	IGHM	Ig mu chain C region	2.76	0.00	-1.36	-0.96
>tr|E9PJK1|E9PJK1_HUMAN Tetraspanin OS=Homo sapiens GN=CD81 PE=1 SV=1; >tr|E9PRJ8|E9PRJ8_HUMAN Tetraspanin (Fragment) OS=Homo sapiens GN=CD81 PE=1 SV=1	CD81	CD81 antigen	4.36	0.00	-1.36	-1.04

Fasta header	Gene name	Protein name	-Log Student's T-test p-value	Student's T-test q-value	Student's T-test Difference	Student's T-test Test statistic
>tr|F8W148|F8W148_HUMAN Carbonic anhydrase 6 (Fragment) OS=Homo sapiens GN=CA6 PE=4 SV=1; >sp|P23280|CAH6_HUMAN Carbonic anhydrase 6 OS=Homo sapiens GN=CA6 PE=1 SV=3	CA6	Carbonic anhydrase 6	2.52	0.00	-1.32	-0.92
>sp|P49327|FAS_HUMAN Fatty acid synthase OS=Homo sapiens GN=FASN PE=1 SV=3	FASN	Fatty acid synthase	3.25	0.00	-1.29	-0.95
>sp|P11021|GRP78_HUMAN 78 kDa glucose-regulated protein OS=Homo sapiens GN=HSPA5 PE=1 SV=2; >sp|Q0VCX2|GRP78_BOVIN 78 kDa glucose-regulated protein OS=Bos taurus GN=HSPA5 PE=2 SV=1	HSPA5	78 kDa glucose-regulated protein	3.10	0.00	-1.29	-0.94
>sp|Q92522|H1X_HUMAN Histone H1x OS=Homo sapiens GN=H1FX PE=1 SV=1	H1FX	Histone H1x	5.42	0.00	-1.28	-1.02
>tr|A0A096LPK4|A0A096LPK4_HUMAN Mucin-5AC OS=Homo sapiens GN=MUC5AC PE=4 SV=1; >sp|P98088|MUC5A_HUMAN Mucin-5AC (Fragments) OS=Homo sapiens GN=MUC5AC PE=1 SV=3	MUC5 AC	Mucin-5AC	2.99	0.00	-1.27	-0.93
>sp|P02788|TRFL_HUMAN Lactotransferrin OS=Homo sapiens GN=LTF PE=1 SV=6; >tr|E7ER44|E7ER44_HUMAN Lactotransferrin OS=Homo sapiens GN=LTF PE=1 SV=1	LTF	Lactotransferrin	5.28	0.00	-1.26	-1.01
>sp|P00747|PLMN_HUMAN Plasminogen OS=Homo sapiens GN=PLG PE=1 SV=2	PLG	Plasminogen	3.94	0.00	-1.23	-0.95
>sp|P62263|RS14_HUMAN 40S ribosomal protein S14 OS=Homo sapiens GN=RPS14 PE=1 SV=3; >tr|H0YB22|H0YB22_HUMAN 40S ribosomal protein S14 (Fragment) OS=Homo sapiens GN=RPS14 PE=1 SV=1	RPS14	40S ribosomal protein S14	3.60	0.00	-1.22	-0.93
>tr|D6RD17|D6RD17_HUMAN Immunoglobulin J chain (Fragment) OS=Homo sapiens GN=IGJ PE=1 SV=3; >sp|P01591|IGJ_HUMAN Immunoglobulin J chain OS=Homo sapiens GN=IGJ PE=1 SV=4	IGJ	Immunoglobulin J chain	1.63	0.01	-1.20	-0.79
>tr|K7EKI8|K7EKI8_HUMAN Periplakin OS=Homo sapiens GN=PPL PE=1 SV=1; >sp|O60437|PEPL_HUMAN Periplakin OS=Homo sapiens GN=PPL PE=1 SV=4	PPL	Periplakin	3.87	0.00	-1.19	-0.92
>sp|P13796|PLSL_HUMAN Plastin-2 OS=Homo sapiens GN=LCP1 PE=1 SV=6; >tr|F1MYX5|F1MYX5_BOVIN Uncharacterized protein OS=Bos taurus GN=LCP1 PE=4 SV=1	LCP1	Plastin-2	5.05	0.00	-1.19	-0.96

Fasta header	Gene name	Protein name	-Log Student's T-test p-value	Student's T-test q-value	Student's T-test Difference	Student's T-test Test statistic
>sp|P68871|HBB_HUMAN Hemoglobin subunit beta OS=Homo sapiens GN=HBB PE=1 SV=2; >tr|F8W6P5|F8W6P5_HUMAN LVV-hemorphin-7 (Fragment) OS=Homo sapiens GN=HBB PE=1 SV=1	HBB	Hemoglobin subunit beta; LVV-hemorphin-7	2.13	0.00	-1.19	-0.83
>sp|P02675|FIBB_HUMAN Fibrinogen beta chain OS=Homo sapiens GN=FGB PE=1 SV=2; >tr|D6REL8|D6REL8_HUMAN Fibrinogen beta chain OS=Homo sapiens GN=FGB PE=1 SV=1	FGB	Fibrinogen beta chain	2.29	0.00	-1.19	-0.84
>sp|Q9UBG3|CRNN_HUMAN Cornulin OS=Homo sapiens GN=CRNN PE=1 SV=1	CRNN	Cornulin	2.54	0.00	-1.16	-0.85
>sp|P31146|COR1A_HUMAN Coronin-1A OS=Homo sapiens GN=CORO1A PE=1 SV=4; >sp|Q92176|COR1A_BOVIN Coronin-1A OS=Bos taurus GN=CORO1A PE=1 SV=3	CORO 1A	Coronin-1A	4.48	0.00	-1.16	-0.92
>sp|Q3T0R1|RS18_BOVIN 40S ribosomal protein S18 OS=Bos taurus GN=RPS18 PE=2 SV=3; >sp|P62269|RS18_HUMAN 40S ribosomal protein S18 OS=Homo sapiens GN=RPS18 PE=1 SV=3	RPS18	40S ribosomal protein S18	4.28	0.00	-1.16	-0.91
>sp|Q8N4F0|BPIB2_HUMAN BPI fold-containing family B member 2 OS=Homo sapiens GN=BPIFB2 PE=1 SV=2	BPIFB2	BPI fold-containing family B member 2	3.56	0.00	-1.15	-0.89
>sp|P02751|FINC_HUMAN Fibronectin OS=Homo sapiens GN=FN1 PE=1 SV=4; >tr|H0Y7Z1|H0Y7Z1_HUMAN Ugl-Y3 (Fragment) OS=Homo sapiens GN=FN1 PE=1 SV=1	FN1	Fibronectin; Ugl-Y3	2.80	0.00	-1.15	-0.85
>sp|P80723|BASP1_HUMAN Brain acid soluble protein 1 OS=Homo sapiens GN=BASP1 PE=1 SV=2	BASP1	Brain acid soluble protein 1	4.81	0.00	-1.14	-0.92
>sp|P31025|LCN1_HUMAN Lipocalin-1 OS=Homo sapiens GN=LCN1 PE=1 SV=1	LCN1	Lipocalin-1	2.31	0.01	-1.08	-0.79
>sp|P02760|AMBP_HUMAN Protein AMBP OS=Homo sapiens GN=AMBP PE=1 SV=1; >tr|S4R471|S4R471_HUMAN Protein AMBP (Fragment) OS=Homo sapiens GN=AMBP PE=4 SV=1	AMBP	Protein AMBP	2.60	0.01	-1.05	-0.79
>sp|P04083|ANXA1_HUMAN Annexin A1 OS=Homo sapiens GN=ANXA1 PE=1 SV=2; >tr|Q5T3N1|Q5T3N1_HUMAN Annexin (Fragment) OS=Homo sapiens GN=ANXA1 PE=1 SV=1	ANXA1	Annexin A1; Annexin	2.62	0.01	-1.05	-0.79
>tr|A1L5B7|A1L5B7_BOVIN SERPINE1 mRNA binding protein 1 OS=Bos taurus GN=SERBP1 PE=2 SV=1;	SERBP 1	Plasminogen activator inhibitor 1 RNA-binding protein	4.31	0.00	-1.04	-0.84

Fasta header	Gene name	Protein name	-Log Student's T-test p-value	Student's T-test q-value	Student's T-test Difference	Student's T-test Test statistic
>sp|Q8NC51|PAIRB_HUMAN Plasminogen activator inhibitor 1 RNA-binding protein OS=Homo sapiens GN=SERBP1 PE=1 SV=2						
>sp|P07357|CO8A_HUMAN Complement component C8 alpha chain OS=Homo sapiens GN=C8A PE=1 SV=2	C8A	Complement component C8 alpha chain	4.34	0.01	-0.96	-0.79

b.

Fasta header	Gene name	Protein name	-Log Student's T-test p-value	Student's T-test q-value	Student's T-test Difference	Student's T-test Test statistic
>sp|P51148|RAB5C_HUMAN Ras-related protein Rab-5C OS=Homo sapiens GN=RAB5C PE=1 SV=2; >sp|Q58DS9|RAB5C_BOVIN Ras-related protein Rab-5C OS=Bos taurus GN=RAB5C PE=2 SV=1	RAB5C	Ras-related protein Rab-5C	4.79	0.01	0.94	0.78
>sp|Q96JP2|MY15B_HUMAN Unconventional myosin-XVB OS=Homo sapiens GN=MYO15B PE=1 SV=2	MYO15 B	Unconventional myosin-XVB	3.88	0.01	0.94	0.77
>tr|F5H3C5|F5H3C5_HUMAN Superoxide dismutase [Mn], mitochondrial (Fragment) OS=Homo sapiens GN=SOD2 PE=1 SV=1; >tr|F5H4R2|F5H4R2_HUMAN Superoxide dismutase [Mn], mitochondrial (Fragment) OS=Homo sapiens GN=SOD2 PE=1 SV=1	SOD2	Superoxide dismutase [Mn], mitochondrial	3.83	0.01	0.95	0.77
>sp|Q9UHR4|BI2L1_HUMAN Brain-specific angiogenesis inhibitor 1-associated protein 2-like protein 1 OS=Homo sapiens GN=BAIAP2L1 PE=1 SV=2	BAIAP 2L1	Brain-specific angiogenesis inhibitor 1-associated protein 2-like protein 1	4.33	0.01	0.96	0.79
>sp|P01009|A1AT_HUMAN Alpha-1-antitrypsin OS=Homo sapiens GN=SERPINA1 PE=1 SV=3	SERPI NA1	Alpha-1-antitrypsin	15.45	0.00	0.96	0.89
>sp|O14745|NHRF1_HUMAN Na(+)/H(+) exchange regulatory cofactor NHE-RF1 OS=Homo sapiens GN=SLC9A3R1 PE=1 SV=4; >tr|J3QRP6|J3QRP6_HUMAN Na(+)/H(+) exchange regulatory cofactor NHE-RF1 (Fragment) OS=Homo sapiens GN=SLC9A3R1 PE=1 SV=1	SLC9A 3R1	Na(+)/H(+) exchange regulatory cofactor NHE-RF1	3.87	0.01	0.97	0.79
>sp|P12277|KCRB_HUMAN Creatine kinase B-type OS=Homo sapiens GN=CKB PE=1 SV=1; >sp|Q5EA61|KCRB_BOVIN Creatine kinase B-type OS=Bos taurus GN=CKB PE=1 SV=1	CKB	Creatine kinase B-type	2.97	0.01	0.98	0.76
>sp|P35705|PRDX3_BOVIN Thioredoxin-dependent peroxide reductase, mitochondrial OS=Bos taurus GN=PRDX3 PE=1 SV=2;	PRDX3	Thioredoxin-dependent peroxide reductase, mitochondrial	3.89	0.01	0.98	0.79

Fasta header	Gene name	Protein name	-Log Student's T-test p-value	Student's T-test q-value	Student's T-test Difference	Student's T-test Test statistic
>sp|P30048|PRDX3_HUMAN Thioredoxin-dependent peroxide reductase, mitochondrial OS=Homo sapiens GN=PRDX3 PE=1 SV=3						
>sp|P02763|A1AG1_HUMAN Alpha-1-acid glycoprotein 1 OS=Homo sapiens GN=ORM1 PE=1 SV=1	ORM1	Alpha-1-acid glycoprotein 1	2.68	0.01	1.01	0.77
>tr|F8VPF3|F8VPF3_HUMAN Myosin light polypeptide 6 (Fragment) OS=Homo sapiens GN=MYL6 PE=4 SV=1; >tr|F8W1R7|F8W1R7_HUMAN Myosin light polypeptide 6 OS=Homo sapiens GN=MYL6 PE=4 SV=1	MYL6	Myosin light polypeptide 6	3.43	0.01	1.01	0.80
>tr|A0A075B6R9|A0A075B6R9_HUMAN Protein IGKV2D-24 (Fragment) OS=Homo sapiens GN=IGKV2D-24 PE=4 SV=1	IGKV2D-24	Protein IGKV2D-24	3.41	0.01	1.02	0.80
>sp|P40926|MDHM_HUMAN Malate dehydrogenase, mitochondrial OS=Homo sapiens GN=MDH2 PE=1 SV=3; >tr|G3XAL0|G3XAL0_HUMAN Malate dehydrogenase OS=Homo sapiens GN=MDH2 PE=1 SV=1	MDH2	Malate dehydrogenase, mitochondrial; Malate dehydrogenase	3.22	0.01	1.02	0.79
>tr|H7C012|H7C012_HUMAN Phospholipase B1, membrane-associated (Fragment) OS=Homo sapiens GN=PLB1 PE=4 SV=1; >tr|H7BYX7|H7BYX7_HUMAN Phospholipase B1, membrane-associated (Fragment) OS=Homo sapiens GN=PLB1 PE=4 SV=1	PLB1	Phospholipase B1, membrane-associated	4.37	0.00	1.02	0.83
>sp|Q8NDH3|PEPL1_HUMAN Probable aminopeptidase NPEPL1 OS=Homo sapiens GN=NPEPL1 PE=1 SV=3; >tr|H0YEP3|H0YEP3_HUMAN Probable aminopeptidase NPEPL1 (Fragment) OS=Homo sapiens GN=NPEPL1 PE=4 SV=1	NPEPL1	Probable aminopeptidase NPEPL1	4.98	0.00	1.02	0.84
>sp|P29622|KAIN_HUMAN Kallistatin OS=Homo sapiens GN=SERPINA4 PE=1 SV=3	SERPINA4	Kallistatin	3.91	0.01	1.02	0.82
>sp|Q8N5I2|ARRD1_HUMAN Arrestin domain-containing protein 1 OS=Homo sapiens GN=ARRDC1 PE=2 SV=1; >tr|Q5T370|Q5T370_HUMAN Arrestin domain-containing protein 1 (Fragment) OS=Homo sapiens GN=ARRDC1 PE=4 SV=1	ARRDC1	Arrestin domain-containing protein 1	3.79	0.01	1.02	0.81
>tr|A0A075B6S3|A0A075B6S3_HUMAN Protein IGKV2-30 (Fragment) OS=Homo sapiens GN=IGKV2-30 PE=4 SV=1; >sp|P06310|KV206_HUMAN Ig kappa chain V-II region RPMI 6410 OS=Homo sapiens PE=4 SV=1	IGKV2-30	Ig kappa chain V-II region RPMI 6410	3.41	0.01	1.04	0.81
>sp|Q9BV40|VAMP8_HUMAN Vesicle-associated membrane protein 8 OS=Homo sapiens GN=VAMP8 PE=1 SV=1; >tr|B8ZZT4|B8ZZT4_HUMAN Vesicle-associated membrane protein 8 OS=Homo sapiens GN=VAMP8 PE=1 SV=1	VAMP8	Vesicle-associated membrane protein 8	4.97	0.00	1.04	0.86
>sp|P15289|ARSA_HUMAN Arylsulfatase A OS=Homo sapiens GN=ARSA PE=1 SV=3	ARSA	Arylsulfatase A	6.15	0.00	1.04	0.88

Fasta header	Gene name	Protein name	-Log Student's T-test p-value	Student's T-test q-value	Student's T-test Difference	Student's T-test Test statistic
>tr|E9PBP6|E9PBP6_HUMAN Microsomal triglyceride transfer protein large subunit OS=Homo sapiens GN=MTTP PE=1 SV=1; >sp|P55157|MTP_HUMAN Microsomal triglyceride transfer protein large subunit OS=Homo sapiens GN=MTTP PE=1 SV=1	MTTP	Microsomal triglyceride transfer protein large subunit	3.78	0.00	1.04	0.83
>sp|P04066|FUCO_HUMAN Tissue alpha-L-fucosidase OS=Homo sapiens GN=FUCA1 PE=1 SV=4	FUCA1	Tissue alpha-L-fucosidase	4.99	0.00	1.05	0.86
>sp|P40879|S26A3_HUMAN Chloride anion exchanger OS=Homo sapiens GN=SLC26A3 PE=1 SV=1	SLC26 A3	Chloride anion exchanger	4.33	0.00	1.06	0.85
>sp|Q12929|EPS8_HUMAN Epidermal growth factor receptor kinase substrate 8 OS=Homo sapiens GN=EPS8 PE=1 SV=1	EPS8	Epidermal growth factor receptor kinase substrate 8	3.65	0.00	1.06	0.84
>sp|Q5E9I6|ARF3_BOVIN ADP-ribosylation factor 3 OS=Bos taurus GN=ARF3 PE=2 SV=3; >sp|P84080|ARF1_BOVIN ADP-ribosylation factor 1 OS=Bos taurus GN=ARF1 PE=1 SV=2	ARF3; ARF1	ADP-ribosylation factor 1;ADP-ribosylation factor 3	4.43	0.00	1.07	0.86
>tr|K7EIG7|K7EIG7_HUMAN Unconventional myosin-Id OS=Homo sapiens GN=MYO1D PE=1 SV=1; >sp|O94832|MYO1D_HUMAN Unconventional myosin-Id OS=Homo sapiens GN=MYO1D PE=1 SV=2	MYO1 D	Unconventional myosin-Id	4.87	0.00	1.08	0.88
>sp|Q6PIF6|MYO7B_HUMAN Unconventional myosin-VIIb OS=Homo sapiens GN=MYO7B PE=1 SV=2	MYO7 B	Unconventional myosin-VIIb	4.43	0.00	1.08	0.87
>sp|Q9Y2T3|GUAD_HUMAN Guanine deaminase OS=Homo sapiens GN=GDA PE=1 SV=1; >tr|Q5SZC6|Q5SZC6_HUMAN Guanine deaminase OS=Homo sapiens GN=GDA PE=1 SV=1	GDA	Guanine deaminase	5.19	0.00	1.08	0.89
>sp|P01040|CYTA_HUMAN Cystatin-A OS=Homo sapiens GN=CSTA PE=1 SV=1; >tr|C9J0E4|C9J0E4_HUMAN Cystatin-A OS=Homo sapiens GN=CSTA PE=1 SV=1	CSTA	Cystatin-A; Cystatin-A, N-terminally processed	3.41	0.00	1.09	0.85
>tr|X6RBG4|X6RBG4_HUMAN Uromodulin OS=Homo sapiens GN=UMOD PE=4 SV=1; >sp|P07911|UROM_HUMAN Uromodulin OS=Homo sapiens GN=UMOD PE=1 SV=1	UMOD	Uromodulin; Uromodulin, secreted form	4.39	0.00	1.10	0.88
>sp|Q9ULC5|ACSL5_HUMAN Long-chain-fatty-acid--CoA ligase 5 OS=Homo sapiens GN=ACSL5 PE=1 SV=1	ACSL5	Long-chain-fatty-acid--CoA ligase 5	4.96	0.00	1.10	0.89
>sp|P21589|5NTD_HUMAN 5-nucleotidase OS=Homo sapiens GN=NT5E PE=1 SV=1; >tr|Q96B60|Q96B60_HUMAN 5-nucleotidase OS=Homo sapiens GN=NT5E PE=2 SV=1	NT5E	5-nucleotidase	4.33	0.00	1.11	0.88

Fasta header	Gene name	Protein name	-Log Student's T-test p-value	Student's T-test q-value	Student's T-test Difference	Student's T-test Test statistic
>sp|P12429|ANXA3_HUMAN Annexin A3 OS=Homo sapiens GN=ANXA3 PE=1 SV=3; >tr|D6RFG5|D6RFG5_HUMAN Annexin (Fragment) OS=Homo sapiens GN=ANXA3 PE=1 SV=1	ANXA3	Annexin A3; Annexin	2.92	0.00	1.12	0.84
>tr|E9PEX6|E9PEX6_HUMAN Dihydrolipoyl dehydrogenase, mitochondrial OS=Homo sapiens GN=DLD PE=1 SV=1; >sp|P09622|DLDH_HUMAN Dihydrolipoyl dehydrogenase, mitochondrial OS=Homo sapiens GN=DLD PE=1 SV=2	DLD	Dihydrolipoyl dehydrogenase, mitochondrial	4.89	0.00	1.16	0.93
>sp|P62833|RAP1A_BOVIN Ras-related protein Rap-1A OS=Bos taurus GN=RAP1A PE=1 SV=1; >sp|P61223|RAP1B_BOVIN Ras-related protein Rap-1b OS=Bos taurus GN=RAP1B PE=2 SV=1	RAP1A; RAP1B	Ras-related protein Rap-1A; Ras-related protein Rap-1b	3.58	0.00	1.18	0.90
>sp|P08582|TRFM_HUMAN Melanotransferrin OS=Homo sapiens GN=MFI2 PE=1 SV=2	MFI2	Melanotransferrin	4.89	0.00	1.18	0.94
>sp|Q07075|AMPE_HUMAN Glutamyl aminopeptidase OS=Homo sapiens GN=ENPEP PE=1 SV=3	ENPEP	Glutamyl aminopeptidase	4.85	0.00	1.20	0.96
>sp|Q14315|FLNC_HUMAN Filamin-C OS=Homo sapiens GN=FLNC PE=1 SV=3; >tr|E1BE25|E1BE25_BOVIN Uncharacterized protein OS=Bos taurus GN=FLNC PE=4 SV=1	FLNC	Filamin-C	6.46	0.00	1.20	0.99
>sp|Q9UBC5|MYO1A_HUMAN Unconventional myosin-Ia OS=Homo sapiens GN=MYO1A PE=1 SV=1; >tr|G3V342|G3V342_HUMAN Unconventional myosin-Ia OS=Homo sapiens GN=MYO1A PE=4 SV=1	MYO1A	Unconventional myosin-Ia	6.18	0.00	1.20	0.99
>sp|Q9H6S3|ES8L2_HUMAN Epidermal growth factor receptor kinase substrate 8-like protein 2 OS=Homo sapiens GN=EPS8L2 PE=1 SV=2	EPS8L2	Epidermal growth factor receptor kinase substrate 8-like protein 2	4.93	0.00	1.21	0.96
>sp|Q8SQH5|ADT2_BOVIN ADP/ATP translocase 2 OS=Bos taurus GN=SLC25A5 PE=2 SV=3; >sp|P05141|ADT2_HUMAN ADP/ATP translocase 2 OS=Homo sapiens GN=SLC25A5 PE=1 SV=7	SLC25A5	ADP/ATP translocase 2; ADP/ATP translocase 2, N-terminally processed	5.21	0.00	1.21	0.98
>sp|P48052|CBPA2_HUMAN Carboxypeptidase A2 OS=Homo sapiens GN=CPA2 PE=1 SV=3	CPA2	Carboxypeptidase A2	2.71	0.00	1.25	0.90
>sp|P45879|VDAC1_BOVIN Voltage-dependent anion-selective channel protein 1 OS=Bos taurus GN=VDAC1 PE=1 SV=3; >tr|F1MIN1|F1MIN1_BOVIN Voltage-dependent anion-selective channel protein 1 OS=Bos taurus GN=VDAC1 PE=4 SV=2	VDAC1	Voltage-dependent anion-selective channel protein 1	5.49	0.00	1.26	1.01
>tr|A0A087WTM7|A0A087WTM7_HUMAN Apolipoprotein B-100 OS=Homo sapiens GN=APOB PE=1 SV=1;	APOB	Apolipoprotein B-100	3.33	0.00	1.28	0.95

Fasta header	Gene name	Protein name	-Log Student's T-test p-value	Student's T-test q-value	Student's T-test Difference	Student's T-test Test statistic
>sp|P04114|APOB_HUMAN Apolipoprotein B-100 OS=Homo sapiens GN=APOB PE=1 SV=2						
>sp|P51884|LUM_HUMAN Lumican OS=Homo sapiens GN=LUM PE=1 SV=2	LUM	Lumican	2.81	0.00	1.30	0.93
>sp|P11678|PERE_HUMAN Eosinophil peroxidase OS=Homo sapiens GN=EPX PE=1 SV=2	EPX	Eosinophil peroxidase	4.21	0.00	1.30	1.00
>sp|P29992|GNA11_HUMAN Guanine nucleotide-binding protein subunit alpha-11 OS=Homo sapiens GN=GNA11 PE=1 SV=2; >sp|P38409|GNA11_BOVIN Guanine nucleotide-binding protein subunit alpha-11 OS=Bos taurus GN=GNA11 PE=2 SV=2	GNA11	Guanine nucleotide-binding protein subunit alpha-11	5.47	0.00	1.31	1.04
>sp|P05543|THBG_HUMAN Thyroxine-binding globulin OS=Homo sapiens GN=SERPINA7 PE=1 SV=2	SERPINA7	Thyroxine-binding globulin	5.47	0.00	1.33	1.06
>tr|C9JFW8|C9JFW8_HUMAN N-acetylated-alpha-linked acidic dipeptidase-like protein OS=Homo sapiens GN=NAALADL1 PE=4 SV=1; >tr|C9JX16|C9JX16_HUMAN N-acetylated-alpha-linked acidic dipeptidase-like protein OS=Homo sapiens GN=NAALADL1 PE=4 SV=2	NAALADL1	N-acetylated-alpha-linked acidic dipeptidase-like protein	6.85	0.00	1.34	1.09
>sp|P28838|AMPL_HUMAN Cytosol aminopeptidase OS=Homo sapiens GN=LAP3 PE=1 SV=3	LAP3	Cytosol aminopeptidase	4.63	0.00	1.34	1.04
>sp|P05154|IPSP_HUMAN Plasma serine protease inhibitor OS=Homo sapiens GN=SERPINA5 PE=1 SV=3; >tr|G3V5Q9|G3V5Q9_HUMAN Plasma serine protease inhibitor (Fragment) OS=Homo sapiens GN=SERPINA5 PE=3 SV=1	SERPINA5	Plasma serine protease inhibitor	3.93	0.00	1.34	1.01
>sp|Q07837|SLC31_HUMAN Neutral and basic amino acid transport protein rBAT OS=Homo sapiens GN=SLC3A1 PE=1 SV=2; >tr|A0A087X0R9|A0A087X0R9_HUMAN Neutral and basic amino acid transport protein rBAT OS=Homo sapiens GN=SLC3A1 PE=4 SV=1	SLC3A1	Neutral and basic amino acid transport protein rBAT	4.97	0.00	1.35	1.05
>tr|A0A087WW89|A0A087WW89_HUMAN Protein IGHV3-72 OS=Homo sapiens GN=IGHV3-72 PE=4 SV=1	IGHV3-72	Protein IGHV3-72	4.37	0.00	1.35	1.03
>sp|P07093|GDN_HUMAN Glia-derived nexin OS=Homo sapiens GN=SERPINE2 PE=1 SV=1	SERPINE2	Glia-derived nexin	5.20	0.00	1.39	1.09
>sp|P10643|CO7_HUMAN Complement component C7 OS=Homo sapiens GN=C7 PE=1 SV=2	C7	Complement component C7	6.34	0.00	1.40	1.12
>tr|K7EMV3|K7EMV3_HUMAN Histone H3 OS=Homo sapiens GN=H3F3B PE=1 SV=1;	H3F3B; H3F3A	Histone H3	3.91	0.00	1.41	1.05

Fasta header	Gene name	Protein name	-Log Student's T-test p-value	Student's T-test q-value	Student's T-test Difference	Student's T-test Test statistic
>tr|B4DEB1|B4DEB1_HUMAN Histone H3 OS=Homo sapiens GN=H3F3A PE=1 SV=1						
>sp|Q9HBB8|CDHR5_HUMAN Cadherin-related family member 5 OS=Homo sapiens GN=CDHR5 PE=1 SV=3	CDHR5	Cadherin-related family member 5	4.95	0.00	1.43	1.10
>sp|Q9BYF1|ACE2_HUMAN Angiotensin-converting enzyme 2 OS=Homo sapiens GN=ACE2 PE=1 SV=2	ACE2	Angiotensin-converting enzyme 2	5.57	0.00	1.43	1.12
>sp|P09210|GSTA2_HUMAN Glutathione S-transferase A2 OS=Homo sapiens GN=GSTA2 PE=1 SV=4; >sp|P08263|GSTA1_HUMAN Glutathione S-transferase A1 OS=Homo sapiens GN=GSTA1 PE=1 SV=3	GSTA2; GSTA1	Glutathione S-transferase A2; Glutathione S-transferase A1	5.51	0.00	1.44	1.13
>tr|H3BMH2|H3BMH2_HUMAN Ras-related protein Rab-11A (Fragment) OS=Homo sapiens GN=RAB11A PE=3 SV=1; >tr|H3BSC1|H3BSC1_HUMAN Ras-related protein Rab-11A OS=Homo sapiens GN=RAB11A PE=1 SV=1	RAB11A	Ras-related protein Rab-11A	6.17	0.00	1.44	1.15
>tr|E7ER45|E7ER45_HUMAN Maltase-glucoamylase, intestinal OS=Homo sapiens GN=MGAM PE=3 SV=2; >sp|O43451|MGA_HUMAN Maltase-glucoamylase, intestinal OS=Homo sapiens GN=MGAM PE=1 SV=5	MGAM	Maltase-glucoamylase, intestinal	5.81	0.00	1.46	1.14
>sp|Q14002|CEAM7_HUMAN Carcinoembryonic antigen-related cell adhesion molecule 7 OS=Homo sapiens GN=CEACAM7 PE=1 SV=1	CEACAM7	Carcinoembryonic antigen-related cell adhesion molecule 7	5.49	0.00	1.46	1.14
>sp|Q6UX06|OLFM4_HUMAN Olfactomedin-4 OS=Homo sapiens GN=OLFM4 PE=1 SV=1	OLFM4	Olfactomedin-4	5.76	0.00	1.48	1.16
>tr|R4GN98|R4GN98_HUMAN Protein S100 (Fragment) OS=Homo sapiens GN=S100A6 PE=1 SV=1; >sp|P06703|S10A6_HUMAN Protein S100-A6 OS=Homo sapiens GN=S100A6 PE=1 SV=1	S100A6	Protein S100; Protein S100-A6	5.41	0.00	1.48	1.15
>sp|Q92820|GGH_HUMAN Gamma-glutamyl hydrolase OS=Homo sapiens GN=GGH PE=1 SV=2	GGH	Gamma-glutamyl hydrolase	5.58	0.00	1.49	1.16
>sp|P62871|GBB1_BOVIN Guanine nucleotide-binding protein G(I)/G(S)/G(T) subunit beta-1 OS=Bos taurus GN=GNB1 PE=1 SV=3; >sp|P62873|GBB1_HUMAN Guanine nucleotide-binding protein G(I)/G(S)/G(T) subunit beta-1 OS=Homo sapiens GN=GNB1 PE=1 SV=3	GNB1	Guanine nucleotide-binding protein G(I)/G(S)/G(T) subunit beta-1	5.58	0.00	1.49	1.16
>sp|P13866|SC5A1_HUMAN Sodium/glucose cotransporter 1 OS=Homo sapiens GN=SLC5A1 PE=1 SV=1	SLC5A1	Sodium/glucose cotransporter 1	6.89	0.00	1.49	1.20
>sp|P12104|FABPI_HUMAN Fatty acid-binding protein, intestinal OS=Homo sapiens GN=FABP2 PE=1 SV=2	FABP2	Fatty acid-binding protein, intestinal	6.82	0.00	1.50	1.20

Fasta header	Gene name	Protein name	-Log Student's T-test p-value	Student's T-test q-value	Student's T-test Difference	Student's T-test Test statistic
>sp|P00915|CAH1_HUMAN Carbonic anhydrase 1 OS=Homo sapiens GN=CA1 PE=1 SV=2; >tr|E5RHP7|E5RHP7_HUMAN Carbonic anhydrase 1 (Fragment) OS=Homo sapiens GN=CA1 PE=1 SV=1	CA1	Carbonic anhydrase 1	3.33	0.00	1.51	1.07
>tr|F5H265|F5H265_HUMAN Polyubiquitin-C (Fragment) OS=Homo sapiens GN=UBC PE=4 SV=1; >tr|J3QS39|J3QS39_HUMAN Ubiquitin (Fragment) OS=Homo sapiens GN=UBB PE=4 SV=1	UBC; UBB	Ubiquitin-60S ribosomal protein L40	5.93	0.00	1.57	1.22
>sp|Q13228|SBP1_HUMAN Selenium-binding protein 1 OS=Homo sapiens GN=SELENBP1 PE=1 SV=2	SELENBP1	Selenium-binding protein 1	4.84	0.00	1.58	1.19
>sp|Q5JS37|NHLC3_HUMAN NHL repeat-containing protein 3 OS=Homo sapiens GN=NHLRC3 PE=2 SV=1; >tr|C9J973|C9J973_HUMAN NHL repeat-containing protein 3 OS=Homo sapiens GN=NHLRC3 PE=4 SV=1	NHLRC3	NHL repeat-containing protein 3	7.60	0.00	1.58	1.27
>sp|P01762|HV301_HUMAN Ig heavy chain V-III region TRO OS=Homo sapiens PE=1 SV=1		Ig heavy chain V-III region TRO	4.05	0.00	1.62	1.17
>sp|P07339|CATD_HUMAN Cathepsin D OS=Homo sapiens GN=CTSD PE=1 SV=1; >tr|F8W787|F8W787_HUMAN Cathepsin D light chain (Fragment) OS=Homo sapiens GN=CTSD PE=3 SV=1	CTSD	Cathepsin D; Cathepsin D light chain	7.08	0.00	1.65	1.30
>sp|P05155|IC1_HUMAN Plasma protease C1 inhibitor OS=Homo sapiens GN=SERPING1 PE=1 SV=2; >tr|E9PGN7|E9PGN7_HUMAN Plasma protease C1 inhibitor OS=Homo sapiens GN=SERPING1 PE=3 SV=1	SERPING1	Plasma protease C1 inhibitor	5.00	0.00	1.66	1.23
>sp|P02748|CO9_HUMAN Complement component C9 OS=Homo sapiens GN=C9 PE=1 SV=2	C9	Complement component C9	5.26	0.00	1.70	1.27
>tr|A0A087X1V9|A0A087X1V9_HUMAN Protein IGKV2-28 OS=Homo sapiens GN=IGKV2-28 PE=4 SV=1; >sp|P01617|KV204_HUMAN Ig kappa chain V-II region TEW OS=Homo sapiens PE=1 SV=1	IGKV2D-28	Ig kappa chain V-II region TEW	4.00	0.00	1.75	1.23
>sp|O75264|SIM24_HUMAN Small integral membrane protein 24 OS=Homo sapiens GN=SMIM24 PE=2 SV=2; >tr|K7EKM7|K7EKM7_HUMAN Small integral membrane protein 24 OS=Homo sapiens GN=SMIM24 PE=4 SV=1	SMIM24	Small integral membrane protein 24	7.45	0.00	1.76	1.38
>sp|Q6UWP2|DHR11_HUMAN Dehydrogenase/reductase SDR family member 11 OS=Homo sapiens GN=DHRS11 PE=1 SV=1; >sp|Q3ZBV9|DHR11_BOVIN Dehydrogenase/reductase SDR family member 11 OS=Bos taurus GN=DHRS11 PE=2 SV=1	DHRS11	Dehydrogenase/reductase SDR family member 11	8.43	0.00	1.79	1.42

Fasta header	Gene name	Protein name	-Log Student's T-test p-value	Student's T-test q-value	Student's T-test Difference	Student's T-test Test statistic
>sp|Q9UBI6|GBG12_HUMAN Guanine nucleotide-binding protein G(I)/G(S)/G(O) subunit gamma-12 OS=Homo sapiens GN=GNG12 PE=1 SV=3	GNG12	Guanine nucleotide-binding protein G(I)/G(S)/G(O) subunit gamma-12	8.50	0.00	1.82	1.44
>sp|P01598|KV106_HUMAN Ig kappa chain V-I region EU OS=Homo sapiens PE=1 SV=1; >tr|A0A075B6S8|A0A075B6S8_HUMAN Ig kappa chain V-I region HK102 (Fragment) OS=Homo sapiens GN=IGKV1-5 PE=4 SV=1	IGKV1-5	Ig kappa chain V-I region EU	5.06	0.00	1.85	1.34
>sp|P55259|GP2_HUMAN Pancreatic secretory granule membrane major glycoprotein GP2 OS=Homo sapiens GN=GP2 PE=2 SV=3	GP2	Pancreatic secretory granule membrane major glycoprotein GP2	6.52	0.00	1.87	1.41
>sp|P15085|CBPA1_HUMAN Carboxypeptidase A1 OS=Homo sapiens GN=CPA1 PE=1 SV=2; >tr|C9JUF9|C9JUF9_HUMAN Carboxypeptidase A1 OS=Homo sapiens GN=CPA1 PE=4 SV=1	CPA1	Carboxypeptidase A1	5.85	0.00	1.88	1.39
>sp|Q92542|NICA_HUMAN Nicastrin OS=Homo sapiens GN=NCSTN PE=1 SV=2; >tr|H0Y6T7|H0Y6T7_HUMAN Nicastrin (Fragment) OS=Homo sapiens GN=NCSTN PE=1 SV=1	NCSTN	Nicastrin	10.39	0.00	1.89	1.53
>sp|P09327|VILI_HUMAN Villin-1 OS=Homo sapiens GN=VIL1 PE=1 SV=4	VIL1	Villin-1	8.25	0.00	1.93	1.50
>sp|P50443|S26A2_HUMAN Sulfate transporter OS=Homo sapiens GN=SLC26A2 PE=1 SV=2	SLC26A2	Sulfate transporter	11.21	0.00	2.01	1.62
>sp|Q6UXC1|AEGP_HUMAN Apical endosomal glycoprotein OS=Homo sapiens GN=MAMDC4 PE=1 SV=2	MAMDC4	Apical endosomal glycoprotein	9.30	0.00	2.02	1.58
>sp|P00390|GSHR_HUMAN Glutathione reductase, mitochondrial OS=Homo sapiens GN=GSR PE=1 SV=2	GSR	Glutathione reductase, mitochondrial	10.27	0.00	2.02	1.61
>sp|P02794|FRIH_HUMAN Ferritin heavy chain OS=Homo sapiens GN=FTH1 PE=1 SV=2; >tr|G3V192|G3V192_HUMAN Ferritin OS=Homo sapiens GN=FTH1 PE=1 SV=1	FTH1	Ferritin heavy chain; Ferritin	6.43	0.00	2.08	1.52
>tr|A6XMV8|A6XMV8_HUMAN Protease serine 2 preproprotein OS=Homo sapiens GN=PRSS3P2 PE=2 SV=1; >tr|A0A096LNX4|A0A096LNX4_HUMAN Putative trypsin-6 OS=Homo sapiens GN=PRSS3P2 PE=4 SV=1	PRSS2	Trypsin-2	5.94	0.00	2.08	1.50
>sp|P09848|LPH_HUMAN Lactase-phlorizin hydrolase OS=Homo sapiens GN=LCT PE=1 SV=3; >tr|H0Y4E4|H0Y4E4_HUMAN Lactase (Fragment) OS=Homo sapiens GN=LCT PE=3 SV=1	LCT	Lactase-phlorizin hydrolase; Lactase	13.66	0.00	2.09	1.73

Fasta header	Gene name	Protein name	-Log Student's T-test p-value	Student's T-test q-value	Student's T-test Difference	Student's T-test Test statistic
>sp|Q9BYE9|CDHR2_HUMAN Cadherin-related family member 2 OS=Homo sapiens GN=CDHR2 PE=1 SV=2	CDHR2	Cadherin-related family member 2	10.00	0.00	2.10	1.65
>sp|P07148|FABPL_HUMAN Fatty acid-binding protein, liver OS=Homo sapiens GN=FABP1 PE=1 SV=1; >tr|A8MW49|A8MW49_HUMAN Fatty acid-binding protein, liver OS=Homo sapiens GN=FABP1 PE=1 SV=1	FABP1	Fatty acid-binding protein, liver	8.51	0.00	2.10	1.61
>sp|Q8TE67|ES8L3_HUMAN Epidermal growth factor receptor kinase substrate 8-like protein 3 OS=Homo sapiens GN=EPS8L3 PE=1 SV=2	EPS8L3	Epidermal growth factor receptor kinase substrate 8-like protein 3	9.61	0.00	2.10	1.64
>sp|Q9NR71|ASAH2_HUMAN Neutral ceramidase OS=Homo sapiens GN=ASAH2 PE=1 SV=2; >tr|E9PBM9|E9PBM9_HUMAN Neutral ceramidase soluble form OS=Homo sapiens GN=ASAH2 PE=4 SV=1	ASAH2; ASAH2C	Neutral ceramidase; Neutral ceramidase soluble form	13.00	0.00	2.11	1.73
>sp|Q03154|ACY1_HUMAN Aminoacylase-1 OS=Homo sapiens GN=ACY1 PE=1 SV=1; >tr|C9JMV9|C9JMV9_HUMAN Protein ABHD14A-ACY1 OS=Homo sapiens GN=ABHD14A-ACY1 PE=4 SV=1	ACY1; ABHD14A-ACY1	Aminoacylase-1	11.79	0.00	2.13	1.72
>sp|P24855|DNAS1_HUMAN Deoxyribonuclease-1 OS=Homo sapiens GN=DNASE1 PE=1 SV=1	DNASE1	Deoxyribonuclease-1	10.81	0.00	2.18	1.73
>sp|P13688|CEAM1_HUMAN Carcinoembryonic antigen-related cell adhesion molecule 1 OS=Homo sapiens GN=CEACAM1 PE=1 SV=2	CEACAM1	Carcinoembryonic antigen-related cell adhesion molecule 1	13.38	0.00	2.25	1.83
>sp|P06731|CEAM5_HUMAN Carcinoembryonic antigen-related cell adhesion molecule 5 OS=Homo sapiens GN=CEACAM5 PE=1 SV=3; >tr|A0A024R0K5|A0A024R0K5_HUMAN Carcinoembryonic antigen-related cell adhesion molecule 5 OS=Homo sapiens GN=CEACAM5 PE=4 SV=1	CEACAM5	Carcinoembryonic antigen-related cell adhesion molecule 5	12.77	0.00	2.33	1.87
>sp|P01717|LV403_HUMAN Ig lambda chain V-IV region Hil OS=Homo sapiens PE=1 SV=1		Ig lambda chain V-IV region Hil	9.82	0.00	2.34	1.79
>sp|Q6UWV6|ENPP7_HUMAN Ectonucleotide pyrophosphatase/phosphodiesterase family member 7 OS=Homo sapiens GN=ENPP7 PE=1 SV=3	ENPP7	Ectonucleotide pyrophosphatase/phosphodiesterase family member 7	12.99	0.00	2.35	1.89
>sp|P20933|ASPG_HUMAN N(4)-(beta-N-acetylglucosaminyl)-L-asparaginase OS=Homo sapiens GN=AGA PE=1 SV=2; >tr|H0Y9C7|H0Y9C7_HUMAN N(4)-(beta-N-acetylglucosaminyl)-L-asparaginase (Fragment) OS=Homo sapiens GN=AGA PE=1 SV=3	AGA	N(4)-(beta-N-acetylglucosaminyl)-L-asparaginase	19.10	0.00	2.40	2.04
>sp|Q9H3R2|MUC13_HUMAN Mucin-13 OS=Homo sapiens GN=MUC13 PE=1 SV=3	MUC13	Mucin-13	7.86	0.00	2.41	1.76

Fasta header	Gene name	Protein name	-Log Student's T-test p-value	Student's T-test q-value	Student's T-test Difference	Student's T-test Test statistic
>sp|P51688|SPHM_HUMAN N-sulphoglucosamine sulphohydrolase OS=Homo sapiens GN=SGSH PE=1 SV=1; >tr|I3NI22|I3NI22_HUMAN N-sulphoglucosamine sulphohydrolase (Fragment) OS=Homo sapiens GN=SGSH PE=4 SV=1	SGSH	N-sulphoglucosamine sulphohydrolase	18.92	0.00	2.44	2.07
>sp|P27216|ANX13_HUMAN Annexin A13 OS=Homo sapiens GN=ANXA13 PE=1 SV=3	ANXA13	Annexin A13	11.22	0.00	2.46	1.91
>sp|Q92485|ASM3B_HUMAN Acid sphingomyelinase-like phosphodiesterase 3b OS=Homo sapiens GN=SMPDL3B PE=2 SV=2; >tr|F8VWW8|F8VWW8_HUMAN Acid sphingomyelinase-like phosphodiesterase 3b OS=Homo sapiens GN=SMPDL3B PE=4 SV=1	SMPDL3B	Acid sphingomyelinase-like phosphodiesterase 3b	13.81	0.00	2.47	1.98
>sp|P01623|KV305_HUMAN Ig kappa chain V-III region WOL OS=Homo sapiens PE=1 SV=1; >sp|P18135|KV312_HUMAN Ig kappa chain V-III region HAH OS=Homo sapiens PE=2 SV=1		Ig kappa chain V-III region WOL; Ig kappa chain V-III region HAH	8.23	0.00	2.48	1.82
>tr|D6RB89|D6RB89_HUMAN Retinol-binding protein 2 (Fragment) OS=Homo sapiens GN=RBP2 PE=3 SV=1; >sp|P50120|RET2_HUMAN Retinol-binding protein 2 OS=Homo sapiens GN=RBP2 PE=1 SV=3	RBP2	Retinol-binding protein 2	15.34	0.00	2.54	2.06
>tr|A0A087WW55|A0A087WW55_HUMAN Trypsin-1 OS=Homo sapiens GN=PRSS1 PE=4 SV=1; >tr|E7EQ64|E7EQ64_HUMAN Trypsin-1 OS=Homo sapiens GN=PRSS1 PE=3 SV=1	PRSS1	Trypsin-1	9.02	0.00	2.59	1.91
>tr|E7EMM4|E7EMM4_HUMAN Acid ceramidase OS=Homo sapiens GN=ASAH1 PE=1 SV=1; >sp|Q13510|ASAH1_HUMAN Acid ceramidase OS=Homo sapiens GN=ASAH1 PE=1 SV=5	ASAH1	Acid ceramidase	16.42	0.00	2.61	2.13
>tr|A0A087WXI2|A0A087WXI2_HUMAN IgGFc-binding protein OS=Homo sapiens GN=FCGBP PE=4 SV=1; >sp|Q9Y6R7|FCGBP_HUMAN IgGFc-binding protein OS=Homo sapiens GN=FCGBP PE=1 SV=3	FCGBP	IgGFc-binding protein	15.33	0.00	2.64	2.13
>sp|P09093|CEL3A_HUMAN Chymotrypsin-like elastase family member 3A OS=Homo sapiens GN=CELA3A PE=1 SV=3	CELA3A	Chymotrypsin-like elastase family member 3A	9.85	0.00	2.64	1.97
>tr|A0A087WYY1|A0A087WYY1_HUMAN Carboxypeptidase Q OS=Homo sapiens GN=CPQ PE=4 SV=1; >sp|Q9Y646|CBPQ_HUMAN Carboxypeptidase Q OS=Homo sapiens GN=CPQ PE=1 SV=1	CPQ	Carboxypeptidase Q	19.09	0.00	2.68	2.24

Fasta header	Gene name	Protein name	-Log Student's T-test p-value	Student's T-test q-value	Student's T-test Difference	Student's T-test Test statistic
>sp|P16444|DPEP1_HUMAN Dipeptidase 1 OS=Homo sapiens GN=DPEP1 PE=1 SV=3	DPEP1	Dipeptidase 1	14.24	0.00	2.69	2.14
>sp|P12821|ACE_HUMAN Angiotensin-converting enzyme OS=Homo sapiens GN=ACE PE=1 SV=1	ACE	Angiotensin-converting enzyme	12.59	0.00	2.70	2.09
>sp|Q04609|FOLH1_HUMAN Glutamate carboxypeptidase 2 OS=Homo sapiens GN=FOLH1 PE=1 SV=1;<br>>sp|Q9HBA9|FOH1B_HUMAN Putative N-acetylated-alpha-linked acidic dipeptidase OS=Homo sapiens GN=FOLH1B PE=2 SV=1	FOLH1;<br>FOLH1B	Glutamate carboxypeptidase 2;<br>Putative N-acetylated-alpha-linked acidic dipeptidase	19.76	0.00	2.75	2.30
>sp|P15144|AMPN_HUMAN Aminopeptidase N OS=Homo sapiens GN=ANPEP PE=1 SV=4	ANPEP	Aminopeptidase N	17.91	0.00	2.78	2.28
>sp|P02743|SAMP_HUMAN Serum amyloid P-component OS=Homo sapiens GN=APCS PE=1 SV=2	APCS	Serum amyloid P-component	17.26	0.00	2.82	2.30
>sp|O60844|ZG16_HUMAN Zymogen granule membrane protein 16 OS=Homo sapiens GN=ZG16 PE=1 SV=2	ZG16	Zymogen granule membrane protein 16	16.02	0.00	2.83	2.27
>sp|P09525|ANXA4_HUMAN Annexin A4 OS=Homo sapiens GN=ANXA4 PE=1 SV=4;<br>>tr|Q6P452|Q6P452_HUMAN Annexin OS=Homo sapiens GN=ANXA4 PE=1 SV=1	ANXA4	Annexin A4;<br>Annexin	14.19	0.00	2.85	2.23
>sp|P02766|TTHY_HUMAN Transthyretin OS=Homo sapiens GN=TTR PE=1 SV=1;<br>>tr|A0A087WT59|A0A087WT59_HUMAN Transthyretin OS=Homo sapiens GN=TTR PE=4 SV=1	TTR	Transthyretin	11.52	0.00	2.91	2.18
>sp|P14410|SUIS_HUMAN Sucrase-isomaltase, intestinal OS=Homo sapiens GN=SI PE=1 SV=6	SI	Sucrase-isomaltase, intestinal	19.66	0.00	2.93	2.42
>sp|P02792|FRIL_HUMAN Ferritin light chain OS=Homo sapiens GN=FTL PE=1 SV=2;<br>>tr|A0A087X1B9|A0A087X1B9_HUMAN Ferritin light chain OS=Homo sapiens GN=FTL PE=1 SV=1	FTL	Ferritin light chain	13.89	0.00	2.94	2.28
>sp|P01625|KV402_HUMAN Ig kappa chain V-IV region Len OS=Homo sapiens PE=1 SV=2;<br>>sp|P06314|KV404_HUMAN Ig kappa chain V-IV region B17 OS=Homo sapiens PE=2 SV=1		Ig kappa chain V-IV region Len;<br>Ig kappa chain V-IV region B17	17.79	0.00	2.94	2.38
>tr|H7C3P4|H7C3P4_HUMAN Glucosamine (N-acetyl)-6-sulfatase (Sanfilippo disease IIID), isoform CRA_b OS=Homo sapiens GN=GNS PE=1 SV=1;<br>>tr|F6S8M0|F6S8M0_HUMAN N-acetylglucosamine-6-sulfatase OS=Homo sapiens GN=GNS PE=1 SV=1	GNS	N-acetylglucosamine-6-sulfatase	23.97	0.00	2.96	2.52
>sp|P00441|SODC_HUMAN Superoxide dismutase [Cu-Zn] OS=Homo sapiens GN=SOD1 PE=1 SV=2;	SOD1	Superoxide dismutase [Cu-Zn]	26.28	0.00	2.99	2.58

Fasta header	Gene name	Protein name	-Log Student's T-test p-value	Student's T-test q-value	Student's T-test Difference	Student's T-test Test statistic
>tr|H7BYH4|H7BYH4_HUMAN Superoxide dismutase [Cu-Zn] OS=Homo sapiens GN=SOD1 PE=1 SV=1						
>tr|A0A075B6I8|A0A075B6I8_HUMAN Protein IGLV1-47 (Fragment) OS=Homo sapiens GN=IGLV1-47 PE=4 SV=1	IGLV1-47	Protein IGLV1-47	17.13	0.00	3.02	2.42
>sp|P35237|SPB6_HUMAN Serpin B6 OS=Homo sapiens GN=SERPINB6 PE=1 SV=3; >tr|A0A024QZX5|A0A024QZX5_HUMAN Serpin B6 OS=Homo sapiens GN=SERPINB6 PE=1 SV=1	SERPINB6	Serpin B6	20.12	0.00	3.04	2.51
>sp|P56470|LEG4_HUMAN Galectin-4 OS=Homo sapiens GN=LGALS4 PE=1 SV=1; >tr|M0QZ93|M0QZ93_HUMAN Galectin (Fragment) OS=Homo sapiens GN=LGALS4 PE=4 SV=1	LGALS4	Galectin-4; Galectin	20.51	0.00	3.37	2.74
>sp|P27487|DPP4_HUMAN Dipeptidyl peptidase 4 OS=Homo sapiens GN=DPP4 PE=1 SV=2	DPP4	Dipeptidyl peptidase 4	16.53	0.00	3.40	2.64
>sp|Q8WWA0|ITLN1_HUMAN Intelectin-1 OS=Homo sapiens GN=ITLN1 PE=1 SV=1	ITLN1	Intelectin-1	53.14	0.00	3.45	3.25
>sp|Q02817|MUC2_HUMAN Mucin-2 OS=Homo sapiens GN=MUC2 PE=1 SV=2	MUC2	Mucin-2	27.59	0.00	3.55	3.02
>tr|B7ZL91|B7ZL91_HUMAN Metalloendopeptidase OS=Homo sapiens GN=MEP1A PE=2 SV=1; >sp|Q16819|MEP1A_HUMAN Meprin A subunit alpha OS=Homo sapiens GN=MEP1A PE=1 SV=2	MEP1A	Metalloendopeptidase; Meprin A subunit alpha	35.26	0.00	3.56	3.16
>sp|Q99895|CTRC_HUMAN Chymotrypsin-C OS=Homo sapiens GN=CTRC PE=1 SV=2	CTRC	Chymotrypsin-C	26.23	0.00	3.74	3.12
>sp|O43895|XPP2_HUMAN Xaa-Pro aminopeptidase 2 OS=Homo sapiens GN=XPNPEP2 PE=1 SV=3	XPNPEP2	Xaa-Pro aminopeptidase 2	33.83	0.00	3.79	3.31
>sp|P08473|NEP_HUMAN Neprilysin OS=Homo sapiens GN=MME PE=1 SV=2	MME	Neprilysin	39.78	0.00	3.99	3.56
>sp|A8K7I4|CLCA1_HUMAN Calcium-activated chloride channel regulator 1 OS=Homo sapiens GN=CLCA1 PE=1 SV=3	CLCA1	Calcium-activated chloride channel regulator 1	21.08	0.00	4.02	3.16
>sp|P09923|PPBI_HUMAN Intestinal-type alkaline phosphatase OS=Homo sapiens GN=ALPI PE=1 SV=2	ALPI	Intestinal-type alkaline phosphatase	44.98	0.00	4.50	4.06

Proteins derived from human or bovine source that were more abundant in (a) gastric aspirates or (b) feces. A Student's T-test difference below zero indicates the protein is higher in abundance in gastric aspirates compared to feces. p-values are -Log transformed. In case protein groups consisted of multiple proteins, the two proteins with highest protein existence (PE) value were selected.

Table S3. Tables of RDA data.

a.

	Df	AIC	F	Pr(>F)
Gestational age	1	388.1	2.648	0.0075 **
Percentage parenteral feeding	1	386	4.092	0.0075 **
Duration first antibiotic treatment	3	387.2	1.506	0.0500 *

b.

	R2.adj	Df	AIC	F	Pr(>F)
Percentage parenteral feeding	0.04668	1	386.3	4.5256	0.002 **
All variables	0.07647				

c.

	RDA1	RDA2
Eigenvalue	15.5138	7.137
Proportion explained	0.5215	0.24
Cumulative proportion	0.5215	0.761

(a) ANOVA table, (b) R_2-adjusted table and (c) Accumulated constrained eigenvalues. Scaling 2 for species and site scores. Species are scaled proportional to eigenvalues. Sites are unscaled: weighted dispersion equal on all dimensions. General scaling constant of scores: 10.99205. **p<0.001, *p< 0.01. p-values are adjusted with False Discovery Rate (FDR).

Table S4. Differentially abundant human- and bovine-derived proteins in feces during the first six postnatal weeks between gestational age groups preterm (25 – 31 weeks of gestation) and term (≥37 weeks of gestation).

a.

Fasta header	Gene name	Protein name	-Log Student's T-test p-value	Student's T-test q-value	Student's T-test Difference	Student's T-test Test statistic
>sp|P01717|LV403_HUMAN Ig lambda chain V-IV region Hil OS=Homo sapiens PE=1 SV=1		Ig lambda chain V-IV region Hil	11.57	0.00	-2.67	-2.03
>tr|H7C3P4|H7C3P4_HUMAN Glucosamine (N-acetyl)-6-sulfatase (Sanfilippo disease IIID), isoform CRA_b OS=Homo sapiens GN=GNS PE=1 SV=1; >tr|F6S8M0|F6S8M0_HUMAN N-acetylglucosamine-6-sulfatase OS=Homo sapiens GN=GNS PE=1 SV=1	GNS	N-acetylglucosamine-6-sulfatase	11.23	0.00	-2.55	-1.95
>sp|P01625|KV402_HUMAN Ig kappa chain V-IV region Len OS=Homo sapiens PE=1 SV=2; >sp|P06314|KV404_HUMAN Ig kappa chain V-IV region B17 OS=Homo sapiens PE=2 SV=1		Ig kappa chain V-IV region Len; Ig kappa chain V-IV region B17	9.17	0.00	-2.23	-1.70
>sp|Q04609|FOLH1_HUMAN Glutamate carboxypeptidase 2 OS=Homo sapiens GN=FOLH1 PE=1 SV=1; >sp|Q9HBA9|FOH1B_HUMAN Putative N-acetylated-alpha-linked acidic dipeptidase OS=Homo sapiens GN=FOLH1B PE=2 SV=1	FOLH1; FOLH1 B	Glutamate carboxypeptidase 2; Putative N-acetylated-alpha-linked acidic dipeptidase	10.03	0.00	-2.18	-1.69
>sp|P80748|LV302_HUMAN Ig lambda chain V-III region LOI OS=Homo sapiens PE=1 SV=1		Ig lambda chain V-III region LOI	4.00	0.01	-1.90	-1.30
>tr|D6RB89|D6RB89_HUMAN Retinol-binding protein 2 (Fragment) OS=Homo sapiens GN=RBP2 PE=3 SV=1; >sp|P50120|RET2_HUMAN Retinol-binding protein 2 OS=Homo sapiens GN=RBP2 PE=1 SV=3	RBP2	Retinol-binding protein 2	5.25	0.01	-1.83	-1.34
>sp|P12821|ACE_HUMAN Angiotensin-converting enzyme OS=Homo sapiens GN=ACE PE=1 SV=1	ACE	Angiotensin-converting enzyme; Angiotensin-converting enzyme, soluble form	9.68	0.01	-1.67	-1.36

b.

Fasta header	Gene name	Protein name	-Log Student's T-test p-value	Student's T-test q-value	Student's T-test Difference	Student's T-test Test statistic
>tr|I3L4Y3|I3L4Y3_HUMAN Pancreatic secretory granule membrane major glycoprotein GP2 (Fragment) OS=Homo sapiens GN=GP2 PE=4 SV=1; >tr|I3L486|I3L486_HUMAN Pancreatic secretory granule membrane major glycoprotein GP2 (Fragment) OS=Homo sapiens GN=GP2 PE=1 SV=1	GP2	Pancreatic secretory granule membrane major glycoprotein GP2 (Fragment)	28.84	0.00	3.00	2.59

Fasta header	Gene name	Protein name	-Log Student's T-test p-value	Student's T-test q-value	Student's T-test Difference	Student's T-test Test statistic
>tr|A0A096LPK4|A0A096LPK4_HUMAN Mucin-5AC OS=Homo sapiens GN=MUC5AC PE=4 SV=1; >sp|P98088|MUC5A_HUMAN Mucin-5AC (Fragments) OS=Homo sapiens GN=MUC5AC PE=1 SV=3	MUC5AC	Mucin-5AC	13.55	0.00	2.71	2.11
>sp|Q86UP6|CUZD1_HUMAN CUB and zona pellucida-like domain-containing protein 1 OS=Homo sapiens GN=CUZD1 PE=2 SV=1	CUZD1	CUB and zona pellucida-like domain-containing protein 1	8.59	0.00	1.86	1.46
>sp|P15086|CBPB1_HUMAN Carboxypeptidase B OS=Homo sapiens GN=CPB1 PE=1 SV=4	CPB1	Carboxypeptidase B	5.25	0.01	1.87	1.36
>tr|D6RD17|D6RD17_HUMAN Immunoglobulin J chain (Fragment) OS=Homo sapiens GN=IGJ PE=1 SV=3; >sp|P01591|IGJ_HUMAN Immunoglobulin J chain OS=Homo sapiens GN=IGJ PE=1 SV=4	IGJ	Immunoglobulin J chain	3.80	0.00	2.22	1.42
>sp|O95497|VNN1_HUMAN Pantetheinase OS=Homo sapiens GN=VNN1 PE=1 SV=2	VNN1	Pantetheinase	7.07	0.00	2.25	1.64
>sp|Q07654|TFF3_HUMAN Trefoil factor 3 OS=Homo sapiens GN=TFF3 PE=1 SV=1; >tr|X6R3S7|X6R3S7_HUMAN Trefoil factor 3 OS=Homo sapiens GN=TFF3 PE=4 SV=2	TFF3	Trefoil factor 3	16.04	0.00	2.48	2.02
>sp|Q14002|CEAM7_HUMAN Carcinoembryonic antigen-related cell adhesion molecule 7 OS=Homo sapiens GN=CEACAM7 PE=1 SV=1	CEACAM 7	Carcinoembryonic antigen-related cell adhesion molecule 7	8.60	0.00	2.66	1.92
>sp|P04118|COL_HUMAN Colipase OS=Homo sapiens GN=CLPS PE=1 SV=2; >tr|A0A087WZW1|A0A087WZW1_HUMAN Colipase OS=Homo sapiens GN=CLPS PE=4 SV=1	CLPS	Colipase	23.83	0.00	3.30	2.72
>sp|Q03403|TFF2_HUMAN Trefoil factor 2 OS=Homo sapiens GN=TFF2 PE=1 SV=2	TFF2	Trefoil factor 2	49.36	0.00	3.64	3.33
>tr|G3V2V8|G3V2V8_HUMAN Epididymal secretory protein E1 (Fragment) OS=Homo sapiens GN=NPC2 PE=1 SV=1; >tr|J3KMY5|J3KMY5_HUMAN Epididymal secretory protein E1 OS=Homo sapiens GN=NPC2 PE=1 SV=1	NPC2	Epididymal secretory protein E1	48.11	0.00	3.83	3.48
>sp|P59666|DEF3_HUMAN Neutrophil defensin 3 OS=Homo sapiens GN=DEFA3 PE=1 SV=1; >sp|P59665|DEF1_HUMAN Neutrophil defensin 1 OS=Homo sapiens GN=DEFA1 PE=1 SV=1	DEFA3; DEFA1	Neutrophil defensin 3; HP 3-56; Neutrophil defensin 1; HP 1-56	31.98	0.00	4.52	3.73
>tr|F8W062|F8W062_HUMAN Phospholipase A2 OS=Homo sapiens GN=PLA2G1B PE=3 SV=1; >sp|P04054|PA21B_HUMAN Phospholipase A2 OS=Homo sapiens GN=PLA2G1B PE=1 SV=3	PLA2G1B	Phospholipase A2	62.78	0.00	4.58	4.26

Fecal proteins derived from human or bovine source that were more abundant in (a) preterm infants or (b) term infants. A Student's T-test difference below zero indicates the protein is higher in abundance in preterm infants compared to term infants. p-values are -Log transformed. In case protein groups consisted of multiple proteins, the two proteins with highest protein existence (PE) value were selected.

Table S5. Human- and bovine-derived proteins identified in more than 50% of the gastric and fecal proteomes of preterm infants (25 – 31 weeks of gestation).

	**Fasta header**
1	>tr|A0A075B6L0|A0A075B6L0_HUMAN Ig lambda-3 chain C regions (Fragment) OS=Homo sapiens GN=IGLC3 PE=4 SV=2;<br>>tr|A0A075B6K9|A0A075B6K9_HUMAN Ig lambda-2 chain C regions (Fragment) OS=Homo sapiens GN=IGLC2 PE=4 SV=1
2	>tr|A0A087WW89|A0A087WW89_HUMAN Protein IGHV3-72 OS=Homo sapiens GN=IGHV3-72 PE=4 SV=1
3	>tr|A0A087WXI2|A0A087WXI2_HUMAN IgGFc-binding protein OS=Homo sapiens GN=FCGBP PE=4 SV=1;<br>>sp|Q9Y6R7|FCGBP_HUMAN IgGFc-binding protein OS=Homo sapiens GN=FCGBP PE=1 SV=3
4	>tr|A0A087WZW8|A0A087WZW8_HUMAN Protein IGKV3-11 OS=Homo sapiens GN=IGKV3-11 PE=4 SV=1
5	>tr|A0A087X0N5|A0A087X0N5_HUMAN Protein IGKV1-17 OS=Homo sapiens GN=IGKV1-17 PE=4 SV=1;<br>>sp|P80362|KV125_HUMAN Ig kappa chain V-I region WAT OS=Homo sapiens PE=1 SV=1
6	>tr|A0A087X2C0|A0A087X2C0_HUMAN Ig mu chain C region OS=Homo sapiens GN=IGHM PE=1 SV=1;<br>>tr|A0A075B6N9|A0A075B6N9_HUMAN Ig mu chain C region (Fragment) OS=Homo sapiens GN=IGHM PE=1 SV=2
7	>tr|C9JF17|C9JF17_HUMAN Apolipoprotein D (Fragment) OS=Homo sapiens GN=APOD PE=4 SV=1;<br>>sp|P05090|APOD_HUMAN Apolipoprotein D OS=Homo sapiens GN=APOD PE=1 SV=1
8	>sp|P00760|TRY1_BOVIN Cationic trypsin OS=Bos taurus PE=1 SV=3;<br>>sp|P00760|TRY1_BOVIN Cationic trypsin OS=Bos taurus GN=Trp1 PE=1 SV=3
9	>sp|P04264|K2C1_HUMAN Keratin, type II cytoskeletal 1 OS=Homo sapiens GN=KRT1 PE=1 SV=6
10	>sp|P08779|K1C16_HUMAN Keratin, type I cytoskeletal 16 OS=Homo sapiens GN=KRT16 PE=1 SV=4
11	>sp|P13645|K1C10_HUMAN Keratin, type I cytoskeletal 10 OS=Homo sapiens GN=KRT10 PE=1 SV=6
12	>sp|P13647|K2C5_HUMAN Keratin, type II cytoskeletal 5 OS=Homo sapiens GN=KRT5 PE=1 SV=3
13	>sp|P35527|K1C9_HUMAN Keratin, type I cytoskeletal 9 OS=Homo sapiens GN=KRT9 PE=1 SV=3
14	>sp|P35908|K22E_HUMAN Keratin, type II cytoskeletal 2 epidermal OS=Homo sapiens GN=KRT2 PE=1 SV=2
15	>tr|G3X807|G3X807_BOVIN Histone H4 (Fragment) OS=Bos taurus PE=3 SV=1;<br>>sp|P62803|H4_BOVIN Histone H4 OS=Bos taurus PE=1 SV=2
16	>tr|F5H265|F5H265_HUMAN Polyubiquitin-C (Fragment) OS=Homo sapiens GN=UBC PE=4 SV=1;<br>>tr|J3QS39|J3QS39_HUMAN Ubiquitin (Fragment) OS=Homo sapiens GN=UBB PE=4 SV=1
17	>tr|G5E5H7|G5E5H7_BOVIN Uncharacterized protein OS=Bos taurus GN=PAEP PE=3 SV=1
18	>sp|P00450|CERU_HUMAN Ceruloplasmin OS=Homo sapiens GN=CP PE=1 SV=1;<br>>tr|E9PFZ2|E9PFZ2_HUMAN Ceruloplasmin OS=Homo sapiens GN=CP PE=4 SV=1
19	>sp|P01008|ANT3_HUMAN Antithrombin-III OS=Homo sapiens GN=SERPINC1 PE=1 SV=1
20	>sp|P01009|A1AT_HUMAN Alpha-1-antitrypsin OS=Homo sapiens GN=SERPINA1 PE=1 SV=3
21	>sp|P01011|AACT_HUMAN Alpha-1-antichymotrypsin OS=Homo sapiens GN=SERPINA3 PE=1 SV=2;<br>>tr|G3V595|G3V595_HUMAN Alpha-1-antichymotrypsin (Fragment) OS=Homo sapiens GN=SERPINA3 PE=4 SV=3

	Fasta header
22	>sp|P01023|A2MG_HUMAN Alpha-2-macroglobulin OS=Homo sapiens GN=A2M PE=1 SV=3
23	>sp|P01024|CO3_HUMAN Complement C3 OS=Homo sapiens GN=C3 PE=1 SV=2
24	>sp|P01833|PIGR_HUMAN Polymeric immunoglobulin receptor OS=Homo sapiens GN=PIGR PE=1 SV=4
25	>sp|P01876|IGHA1_HUMAN Ig alpha-1 chain C region OS=Homo sapiens GN=IGHA1 PE=1 SV=2
26	>sp|P02768|ALBU_HUMAN Serum albumin OS=Homo sapiens GN=ALB PE=1 SV=2;<br>>tr|B7WNR0|B7WNR0_HUMAN Serum albumin OS=Homo sapiens GN=ALB PE=1 SV=1
27	>sp|P02787|TRFE_HUMAN Serotransferrin OS=Homo sapiens GN=TF PE=1 SV=3
28	>sp|P02788|TRFL_HUMAN Lactotransferrin OS=Homo sapiens GN=LTF PE=1 SV=6;<br>>tr|E7ER44|E7ER44_HUMAN Lactotransferrin OS=Homo sapiens GN=LTF PE=1 SV=1
29	>sp|P02794|FRIH_HUMAN Ferritin heavy chain OS=Homo sapiens GN=FTH1 PE=1 SV=2;<br>>tr|G3V192|G3V192_HUMAN Ferritin OS=Homo sapiens GN=FTH1 PE=1 SV=1
30	>sp|P04745|AMY1_HUMAN Alpha-amylase 1 OS=Homo sapiens GN=AMY1A PE=1 SV=2;<br>>sp|P19961|AMY2B_HUMAN Alpha-amylase 2B OS=Homo sapiens GN=AMY2B PE=1 SV=1
31	>sp|P05109|S10A8_HUMAN Protein S100-A8 OS=Homo sapiens GN=S100A8 PE=1 SV=1
32	>sp|P05155|IC1_HUMAN Plasma protease C1 inhibitor OS=Homo sapiens GN=SERPING1 PE=1 SV=2;<br>>tr|E9PGN7|E9PGN7_HUMAN Plasma protease C1 inhibitor OS=Homo sapiens GN=SERPING1 PE=3 SV=1
33	>sp|P05164|PERM_HUMAN Myeloperoxidase OS=Homo sapiens GN=MPO PE=1 SV=1
34	>sp|P06702|S10A9_HUMAN Protein S100-A9 OS=Homo sapiens GN=S100A9 PE=1 SV=1
35	>sp|P07355|ANXA2_HUMAN Annexin A2 OS=Homo sapiens GN=ANXA2 PE=1 SV=2;<br>>tr|H0YN42|H0YN42_HUMAN Annexin (Fragment) OS=Homo sapiens GN=ANXA2 PE=1 SV=1
36	>sp|P08727|K1C19_HUMAN Keratin, type I cytoskeletal 19 OS=Homo sapiens GN=KRT19 PE=1 SV=4;<br>>tr|C9JM50|C9JM50_HUMAN Keratin, type I cytoskeletal 19 (Fragment) OS=Homo sapiens GN=KRT19 PE=1 SV=1
37	>sp|P15085|CBPA1_HUMAN Carboxypeptidase A1 OS=Homo sapiens GN=CPA1 PE=1 SV=2;<br>>tr|C9JUF9|C9JUF9_HUMAN Carboxypeptidase A1 OS=Homo sapiens GN=CPA1 PE=4 SV=1
38	>sp|P15144|AMPN_HUMAN Aminopeptidase N OS=Homo sapiens GN=ANPEP PE=1 SV=4
39	>sp|P25311|ZA2G_HUMAN Zinc-alpha-2-glycoprotein OS=Homo sapiens GN=AZGP1 PE=1 SV=2;<br>>tr|C9JEV0|C9JEV0_HUMAN Zinc-alpha-2-glycoprotein OS=Homo sapiens GN=AZGP1 PE=3 SV=1
40	>sp|P30740|ILEU_HUMAN Leukocyte elastase inhibitor OS=Homo sapiens GN=SERPINB1 PE=1 SV=1
41	>sp|P47989|XDH_HUMAN Xanthine dehydrogenase/oxidase OS=Homo sapiens GN=XDH PE=1 SV=4
42	>sp|P55259|GP2_HUMAN Pancreatic secretory granule membrane major glycoprotein GP2 OS=Homo sapiens GN=GP2 PE=2 SV=3
43	>sp|P60174|TPIS_HUMAN Triosephosphate isomerase OS=Homo sapiens GN=TPI1 PE=1 SV=3;<br>>sp|Q5E956|TPIS_BOVIN Triosephosphate isomerase OS=Bos taurus GN=TPI1 PE=2 SV=3

	Fasta header
44	>sp|P63258|ACTG_BOVIN Actin, cytoplasmic 2 OS=Bos taurus GN=ACTG1 PE=1 SV=1;<br>>sp|P63261|ACTG_HUMAN Actin, cytoplasmic 2 OS=Homo sapiens GN=ACTG1 PE=1 SV=1
45	>sp|Q08380|LG3BP_HUMAN Galectin-3-binding protein OS=Homo sapiens GN=LGALS3BP PE=1 SV=1;<br>>tr|K7EP36|K7EP36_HUMAN Galectin-3-binding protein (Fragment) OS=Homo sapiens GN=LGALS3BP PE=1 SV=1
46	>sp|Q13228|SBP1_HUMAN Selenium-binding protein 1 OS=Homo sapiens GN=SELENBP1 PE=1 SV=2
47	>sp|Q9UGM3|DMBT1_HUMAN Deleted in malignant brain tumors 1 protein OS=Homo sapiens GN=DMBT1 PE=1 SV=2

In case protein groups consisted of multiple proteins, the two proteins with highest protein existence (PE) value were selected.

Table S6. Bacterial oxidative stress proteins from opportunistic pathogens including *Enterococcus* spp., *Escherichia* spp. and *Klebsiella* spp.

	Fasta header
1	>tr|D5CGU0|D5CGU0_ENTCC Superoxide dismutase OS=Enterobacter cloacae subsp. cloacae (strain ATCC 13047 / DSM 30054 / NBRC 13535 / NCDC 279-56) GN=ECL_05067 PE=3 SV=1;<br>>tr|A0A023V7A4|A0A023V7A4_CITFR Superoxide dismutase OS=Citrobacter freundii CFNIH1 GN=CFN
2	>tr|A6LCS3|A6LCS3_PARD8 Catalase OS=Parabacteroides distasonis (strain ATCC 8503 / DSM 20701 / NCTC 11152) GN=BDI_1740 PE=3 SV=1;<br>>tr|Q5LG24|Q5LG24_BACFN Catalase OS=Bacteroides fragilis (strain ATCC 25285 / NCTC 9343) GN=katA PE=3 SV=1
3	>tr|A6T7U8|A6T7U8_KLEP7 Catalase OS=Klebsiella pneumoniae subsp. pneumoniae (strain ATCC 700721 / MGH 78578) GN=katE PE=3 SV=1
4	>sp|A6T9H9|KATG_KLEP7 Catalase-peroxidase OS=Klebsiella pneumoniae subsp. pneumoniae (strain ATCC 700721 / MGH 78578) GN=katG PE=3 SV=1
5	>tr|A6TA04|A6TA04_KLEP7 Superoxide dismutase OS=Klebsiella pneumoniae subsp. pneumoniae (strain ATCC 700721 / MGH 78578) GN=sodB PE=3 SV=1;<br>>tr|D5CBR7|D5CBR7_ENTCC Superoxide dismutase OS=Enterobacter cloacae subsp. cloacae (strain ATCC 13047 / DSM 30054 /
6	>tr|D5CC12|D5CC12_ENTCC Catalase OS=Enterobacter cloacae subsp. cloacae (strain ATCC 13047 / DSM 30054 / NBRC 13535 / NCDC 279-56) GN=ECL_02433 PE=3 SV=1;<br>>sp|P21179|CATE_ECOLI Catalase HPII OS=Escherichia coli (strain K12) GN=katE PE=1 SV=1
7	>tr|V5VFT2|V5VFT2_ACIBA Catalase OS=Acinetobacter baumannii GN=P795_10275 PE=4 SV=1;<br>>tr|F0KKY1|F0KKY1_ACICP Hydroperoxidase II OS=Acinetobacter calcoaceticus (strain PHEA-2) GN=katE PE=4 SV=1
8	>sp|P0A0J3|SODM1_STAA8 Superoxide dismutase [Mn] 1 OS=Staphylococcus aureus (strain NCTC 8325) GN=sodA PE=1 SV=1
9	>sp|P0AE08|AHPC_ECOLI Alkyl hydroperoxide reductase subunit C OS=Escherichia coli (strain K12) GN=ahpC PE=1 SV=2;<br>>tr|Q32IW2|Q32IW2_SHIDS Alkyl hydroperoxide reductase, C22 subunit OS=Shigella dysenteriae serotype 1 (strain Sd197) GN=ahpC PE=4 SV=1
10	>tr|Q32FB5|Q32FB5_SHIDS Superoxide dismutase OS=Shigella dysenteriae serotype 1 (strain Sd197) GN=sodB PE=3 SV=1;<br>>sp|P0AGD3|SODF_ECOLI Superoxide dismutase [Fe] OS=Escherichia coli (strain K12) GN=sodB PE=1 SV=2
11	>sp|P13029|KATG_ECOLI Catalase-peroxidase OS=Escherichia coli (strain K12) GN=katG PE=1 SV=2
12	>sp|Q2FYU7|CATA_STAA8 Catalase OS=Staphylococcus aureus (strain NCTC 8325) GN=katA PE=2 SV=2
13	>sp|Q5HNZ5|SODM_STAEQ Superoxide dismutase [Mn/Fe] OS=Staphylococcus epidermidis (strain ATCC 35984 / RP62A) GN=sodA PE=3 SV=1
14	>sp|Q5HPK8|CATA_STAEQ Catalase OS=Staphylococcus epidermidis (strain ATCC 35984 / RP62A) GN=katA PE=3 SV=1
15	>sp|Q5HRY1|AHPC_STAEQ Alkyl hydroperoxide reductase subunit C OS=Staphylococcus epidermidis (strain ATCC 35984 / RP62A) GN=ahpC PE=3 SV=1
16	>sp|Q838I4|SODM_ENTFA Superoxide dismutase [Fe] OS=Enterococcus faecalis (strain ATCC 700802 / V583) GN=sodA PE=3 SV=1
17	>sp|P37689|GPMI_ECOLI 2,3-bisphosphoglycerate-independent phosphoglycerate mutase OS=Escherichia coli (strain K12) GN=gpmI PE=1 SV=1
18	>sp|P77212|RCLA_ECOLI Probable pyridine nucleotide-disulfide oxidoreductase RclA OS=Escherichia coli (strain K12) GN=rclA PE=2 SV=2
19	>tr|Q838J4|Q838J4_ENTFA OsmC/Ohr family protein OS=Enterococcus faecalis (strain ATCC 700802 / V583) GN=EF_0453 PE=4 SV=1

In case protein groups consisted of multiple proteins, the first proteins with highest protein existence (PE) value was selected.
